# Supplementary material for: Supramolecular assembly activated single-molecule phosphorescence resonance energy transfer for near-infrared targeted cell imaging
Source: Nat Commun. 2024 Jun 5;15:4787. doi: 10.1038/s41467-024-49238-5 (PMC11153566; doi:10.1038/s41467-024-49238-5)
Supplement: Supplementary file 1 — Supplementary Information [file 41467_2024_49238_MOESM1_ESM.pdf]

Supplementary Information

Supramolecular Assembly Activated Single-Molecule Phosphorescence Resonance Energy Transfer for Near-Infrared Targeted Cell Imaging

Xiaolu Zhou†[1], Xue Bai†[1], Fangjian Shang[1], Heng-Yi Zhang[1], Li-Hua Wang[1], Xiufang Xu[1] and Yu Liu[1,2]*

[1]College of Chemistry, State Key Laboratory of Elemento-Organic Chemistry, Nankai University, Tianjin 300071, P. R. China.

[2]Collaborative Innovation Center of Chemical Science and Engineering (Tianjin), Nankai University, Tianjin 300071, P. R. China.

1. Supplementary Methods.

1.1 Theoretical calculation

Geometry optimization of TPE-DPY, PY-1, supramolecular assembly PY-1/CB[8] and TPE-1/CB[7] were performed in Gaussian 16, Revision C.02 program[1] using M06-2X[2] functional with D3 dispersion correction[3] and 6-31G(d) basis set with SMD model (water as solvent)[4]. The single-point calculations were further improved by using M06-2X[2] functional together with a basis set of def2-TZVP[5] with SMD model (water as solvent) on the optimized geometries. The TD-DFT and SOC results were given by using M06-2X functional with D3 dispersion correction and def2-SV(P)[5] basis set (def2/J[6] as auxiliary basis) together with RIJCOSX algorithm[7] and SMD model (water as solvent) based on optimized S_0 geometry. Single point energy calculations were carried out using Gaussian 16 program. SOC and TD-DFT calculations were carried out using the quantum chemistry program ORCA[8] version 5.0.4. The orbital diagram was performed using Multiwfn 3.8[9] and GaussView 6.0 program.

1.2 Synthesis and Characterization

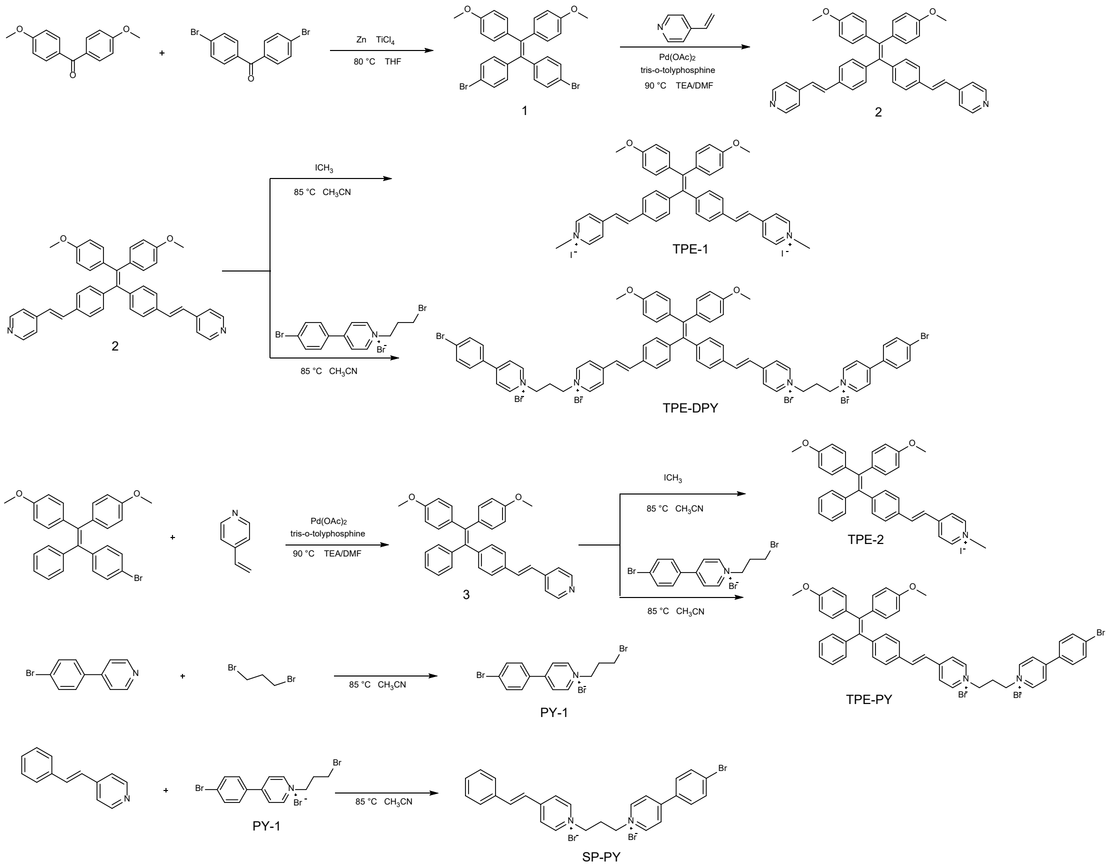

Supplementary Fig. 1 Synthetic route of TPE-DPY, TPE-PY, TPE-1, TPE-2, PY-1 and SP-PY.

Synthesis of compounds 1:

Compound 1 was synthesized according to the literature.[10] 4,4' -dibromobenzophenone (696 mg, 2.06 mmol), 4,4' -dimethoxybenzophenone (500 mg, 2.06 mmol) and Zinc powder (1.35 g, 21 mmol) were added to anhydrous THF (15ml). The mixture was evacuated and refilled with argon gas three times and then cooled to 0°C in an ice bath. $TiCl_4$ (1.2 ml, 10.3mmol) was added to the mixture drop by drop, then the reaction mixture was heated to 80°C and kept in reflux overnight. After the solvent was evaporated, the crude product was further purified by column chromatography with petroleum ether-ethyl acetate (10:1) as an eluent to afford compound 1 as a yellow powder (400 mg, 35.4%). ^{1}H NMR (400 MHz, DMSO-d_6) δ 7.35 (d, J = 8.5 Hz, 4H), 6.91 – 6.84 (m, 8H), 6.73 (d, J = 8.8 Hz, 4H), 3.69 (s, 6H).

Synthesis of compounds 2:

Palladium acetate (9 mg, 0.04 mmol), tris-o-tolylphosphine (24 mg, 0.08 mmol), compound 1 (220 mg, 0.4 mmol) and 4-vinylpyridine (92 mg, 0.88 mmol) were introduced into a 25 ml dry flask with TEA/DMF (15 ml, 2:1, v/v) mixture, which was stirred and degassed for 15 min. The system was stirred at 85 °C under argon for 24 h. The crude was cooled to room temperature, TEA was removed by rotary evaporation, and the mixture was diluted with dichloromethane and then washed with saturated $NaHCO_3$ and water. The obtained mixture was dry with anhydrous $MgSO_4$ overnight. Then, the crude product was further purified by column chromatography with petroleum ether-ethyl acetate (1:1) as an eluent to afford compound 2 as a yellow powder (153 mg, yield: 64%). ^{1}H NMR (400 MHz, DMSO-d_6) δ 8.53 (d, J = 6.1 Hz, 4H), 7.53 – 7.50 (m, 4H), 7.48 – 7.42 (m, 6H), 7.18 (d, J = 16.5 Hz, 2H), 7.00 (d, J = 8.1 Hz, 4H), 6.91 (d, J = 8.7 Hz, 4H), 6.73 (d, J = 8.7 Hz, 4H), 3.69 (s, 6H).

Synthesis of compound 3:

4,4'-(2-(4-bromophenyl)-2-phenylethene-1,1-diyl)bis(methoxybenzene) (500 mg, 1.06 mmol), 4-vinylpyridine (136 mg, 1.3 mmol), Palladium acetate (24 mg, 0.11mmol), and tris-o-tolylphosphine (67 mg, 0.22 μmol) were introduced into a 25 ml dry flask with TEA/DMF (15 ml, 2:1, v/v) mixture, which was stirred and degassed for 15 min. The system was stirred at 85 °C under argon for 24 h. The crude was cooled to room

temperature, TEA was removed by rotary evaporation, and the mixture was diluted with dichloromethane and then washed with saturated $NaHCO_3$ and water. The obtained mixture was dry with anhydrous $MgSO_4$ overnight. Then, the crude product was further purified by column chromatography with petroleum ether-ethyl acetate (5:1) as an eluent to afford compound 3 as a yellow powder (346 mg, yield: 66%). ^{1}H NMR (400 MHz, DMSO-d_6) δ 8.53 (d, J = 6.1 Hz, 2H), 7.54 – 7.49 (m, 2H), 7.48 – 7.40 (m, 3H), 7.19 (d, J = 3.4 Hz, 1H), 7.18 – 7.14 (m, 2H), 7.13 (d, J = 7.0 Hz, 1H), 7.01 – 6.96 (m, 4H), 6.91 (d, J = 8.8 Hz, 2H), 6.87 (d, J = 8.8 Hz, 2H), 6.73 (d, J = 8.8 Hz, 2H), 6.70 (d, J = 8.8 Hz, 2H), 3.69 (d, J = 2.0 Hz, 6H).

Synthesis of compound TPE-PY:

Compound 3 (18 mg, 0.04 mmol) and PY-1 (13 mg, 0.03 mmol) were added into CH_3CN (5 ml). The reaction mixture was heated to 85 °C and refluxed under N_2 atmosphere for 48 hours. Then, the mixture was filtered, and the obtained solid was washed with cold acetonitrile and ethyl acetate three times. Finally, the product (16.7 mg, yield: 60%) was dried in a vacuum dryer overnight as an orange powder. ^{1}H NMR (400 MHz, Methanol-d_4) δ 9.04 (d, J = 6.9 Hz, 2H), 8.82 (d, J = 6.7 Hz, 2H), 8.45 (d, J = 6.9 Hz, 2H), 8.16 (d, J = 6.7 Hz, 2H), 7.94 (d, J = 8.7 Hz, 2H), 7.86 (d, J = 16.2 Hz, 1H), 7.81 (d, J = 8.7 Hz, 2H), 7.49 (d, J = 8.2 Hz, 2H), 7.34 (d, J = 16.2 Hz, 1H), 7.12 (d, J = 7.3 Hz, 3H), 7.08 (d, J = 8.4 Hz, 2H), 7.00 (dd, J = 7.6, 1.9 Hz, 2H), 6.92 (dd, J = 15.5, 8.8 Hz, 4H), 6.66 (dd, J = 12.1, 8.8 Hz, 4H), 4.84 – 4.78 (m, 2H), 4.73 (t, J = 7.7 Hz, 2H), 3.71 (s, 6H), 2.78 (p, J = 7.8 Hz, 2H).; ^{13}C NMR (101 MHz, Methanol-d_4) δ 158.74, 158.60, 155.82, 154.72, 147.58, 144.79, 143.89, 143.81, 141.97, 141.86, 138.40, 135.88, 135.76, 132.80, 132.34, 132.26, 131.86, 131.09, 129.58, 127.58, 127.04, 126.14, 123.93, 121.88, 112.88, 112.71, 57.27, 56.76, 54.20, 31.94.; HRMS (ESI) m/z for $C_{49}H_{43}Br_3N_2O_2$ calcd. $[M-2Br]^{2+}$ 385.1249, found: 385.1244.

Synthesis of reference compound TPE-1, TPE-2, PY-1 and SP-PY:

Synthesis of TPE-1: Compound 2 (50 mg, 0.08 mmol) and ICH_3 (0.21 mmol, 13 µl, ρ=2.28 g/cm^3) were dissolved in CH_3CN (5 mL). The mixture was heated to 85 °C under an argon atmosphere and refluxed for 48 h. Subsequently, the mixture was filtered and the residue was washed with sonication by ethyl acetate and ether three

times, respectively. The product (35 mg, yield: 49%) was obtained after being dried in a vacuum overnight. ^{1}H NMR (400 MHz, DMSO-d_6) δ 8.83 (d, J = 6.6 Hz, 4H), 8.16 (d, J = 6.5 Hz, 4H), 7.91 (d, J = 16.4 Hz, 2H), 7.55 (d, J = 8.1 Hz, 4H), 7.44 (d, J = 16.3 Hz, 2H), 7.08 (d, J = 8.2 Hz, 4H), 6.92 (d, J = 8.6 Hz, 4H), 6.74 (d, J = 8.7 Hz, 4H), 4.24 (s, 6H), 3.69 (s, 6H).

Synthesis of TPE-2: Similar to the synthesis of compound TPE-1, compound 3 (164 mg, 0.33 mmol) and ICH_3 (0.51 mmol, 32 μl, ρ=2.28 g/cm^3) were dissolved in CH_3CN (5 mL). The mixture was heated to 85 °C under an argon atmosphere for 48 h. After the reaction was completed, the mixture was filtered and ultrasonic washed with ethyl acetate and ether three times, respectively. The product (44.31 mg, yield: 21%) was obtained after being dried in a vacuum overnight. ^{1}H NMR (400 MHz, Methanol-d_4) δ 8.66 (d, J = 6.8 Hz, 2H), 8.09 (d, J = 6.7 Hz, 2H), 7.82 (d, J = 16.2 Hz, 1H), 7.49 (d, J = 8.3 Hz, 2H), 7.32 (d, J = 16.2 Hz, 1H), 7.12 (d, J = 7.4 Hz, 3H), 7.07 (d, J = 8.3 Hz, 2H), 7.00 (dd, J = 7.6, 1.9 Hz, 2H), 6.94 (d, J = 8.8 Hz, 2H), 6.90 (d, J = 8.8 Hz, 2H), 6.67 (d, J = 8.8 Hz, 2H), 6.65 (d, J = 8.8 Hz, 2H), 4.28 (s, 3H), 3.71 (d, J = 0.9 Hz, 6H).

Synthesis of PY-1: 4-(4-bromophenyl)pyridine (100 mg, 0.43 mmol) and 1,3-dibromopropane (0.35 g, 1.75 mmol) were dissolved in 25 mL CH_3CN and heated at 60 °C for 12 h. After being cooled to room temperature, the reaction mixture was dispersed in 250 mL diethyl ether. The mixture was filtered and the solid was washed with acetone. The product was obtained as a white solid (120 mg, 65%). ^{1}H NMR (400 MHz, DMSO-d_6) δ 9.12 (d, J = 7.0 Hz, 2H), 8.55 (d, J = 7.0 Hz, 2H), 8.05 (d, J = 8.7 Hz, 2H), 7.88 (d, J = 8.7 Hz, 2H), 4.69 (t, J = 7.1 Hz, 2H), 3.60 (t, J = 6.6 Hz, 2H), 2.55 (d, J = 6.9 Hz, 2H).

Synthesis of SP-PY: Similar to the synthesis of compound TPE-PY, 4-styrylpyridine (13 mg, 0.07 mmol) and PY-1 (22 mg, 0.05 mmol) were added into CH_3CN (5 ml). The reaction mixture was heated to 85 °C and refluxed under N_2 atmosphere for 48 hours. Then, the mixture was filtered, and the obtained solid was washed with cold acetonitrile and ethyl acetate three times. Finally, the product (25 mg, yield: 82%) was dried in a vacuum dryer overnight as a yellow powder. ^{1}H NMR (400 MHz, DMSO-d_6) δ 9.11 (d, J = 6.3 Hz, 2H), 8.95 (d, J = 6.2 Hz, 2H), 8.59 (d, J = 6.1 Hz, 2H), 8.29 (d, J = 6.1 Hz,

2H), 8.08 – 8.01 (m, 3H), 7.88 (d, J = 8.5 Hz, 2H), 7.76 (d, J = 7.4 Hz, 2H), 7.59 – 7.48 (m, 4H), 4.66 (d, J = 25.0 Hz, 4H), 2.68 – 2.66 (m, 2H).

Synthesis of HACD: The HACD that we used was synthesized based on the literature,[11] the degree of substitution (DS) was calculated by the ^{1}H NMR integral of the corresponding characteristic peaks. 1-Ethyl-3-(3-dimethyl aminopropyl)-carbodiimide hydrochloride (419 mg, 2.2 mmol), N-hydroxysuccinimide (251 mg, 2.2 mmol) was added to a solution of sodium hyaluronate (Mw = 93000) (250 mg) in phosphate buffer solution (PBS, 0.1 M, pH 7.2) (150 mL). Then, mono-6-deoxy-6-ethylenediamino-β-CD (2700mg, 2.3 mmol) in PBS (50 mL) was added to the above solution with vigorous stirring and reacted for 28 h at room temperature. The resulting solution was dialyzed with an excess amount of water for 5 days. HACD was obtained as a white solid after freeze-dried. ^{1}H NMR (400 MHz, D_2O) δ 4.99 (m, 2.35H), 4.39 (d, 1.95H), 3.90 – 3.15 (m, 27.47H), 1.93 (s, 2.89H).

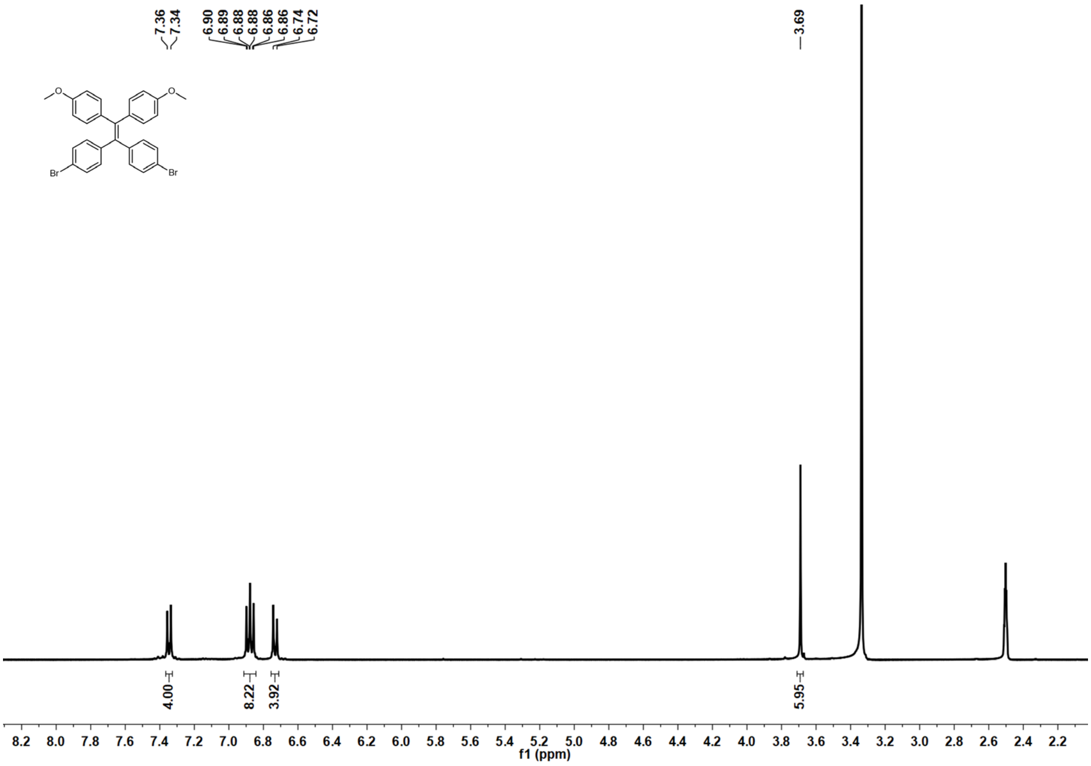

Supplementary Fig. 2 ^{1}H NMR spectrum of compound 1(DMSO-d_6, 400 MHz, 298K).

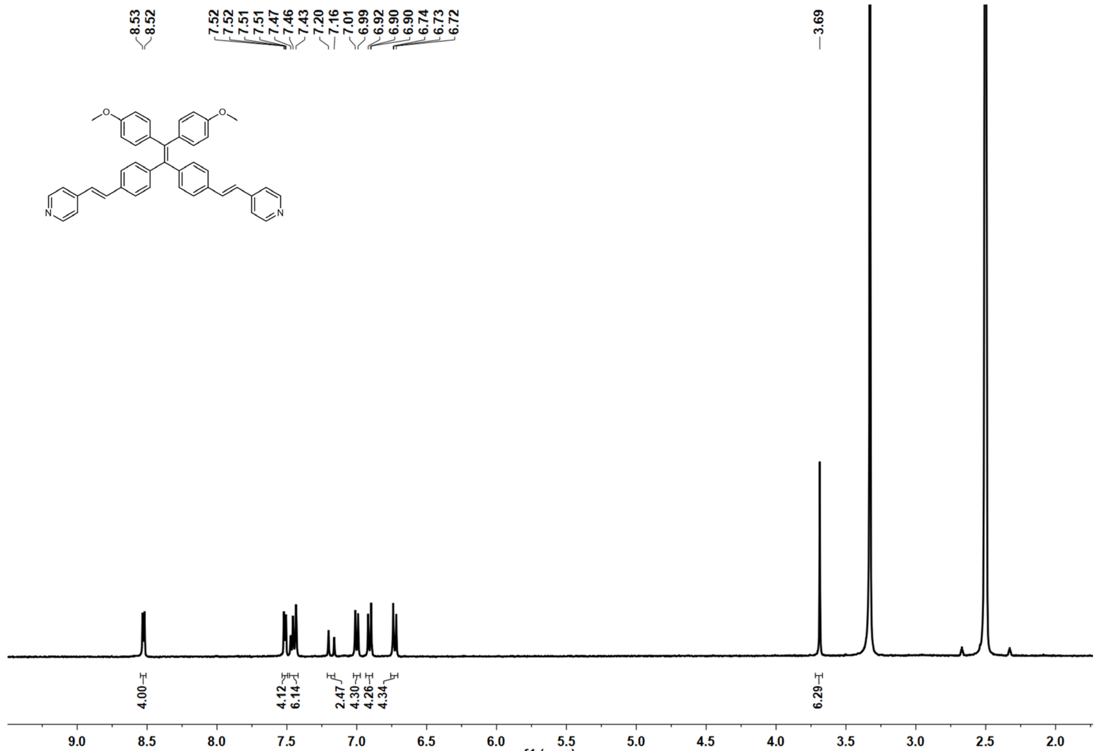

Supplementary Fig. 3 ^{1}H NMR spectrum of compound 2(DMSO-d_6, 400 MHz, 298K).

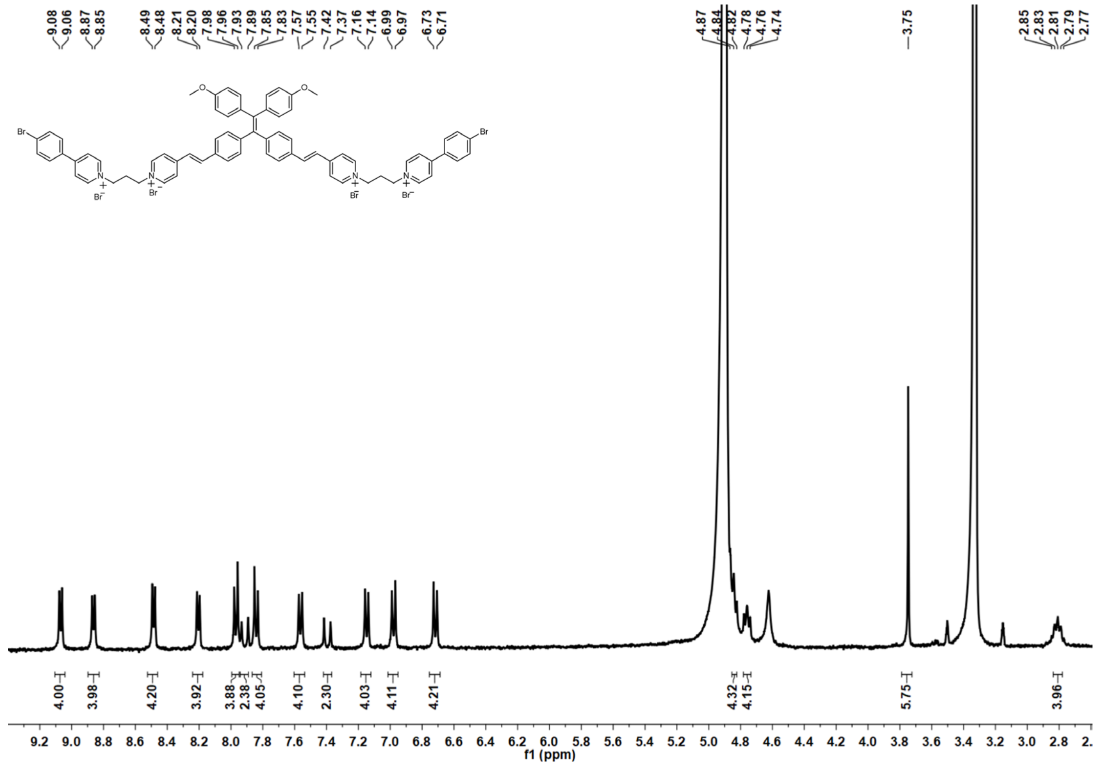

Supplementary Fig. 4 ^{1}H NMR spectrum of TPE-DPY (Methanol-d_4, 400 MHz, 298K).

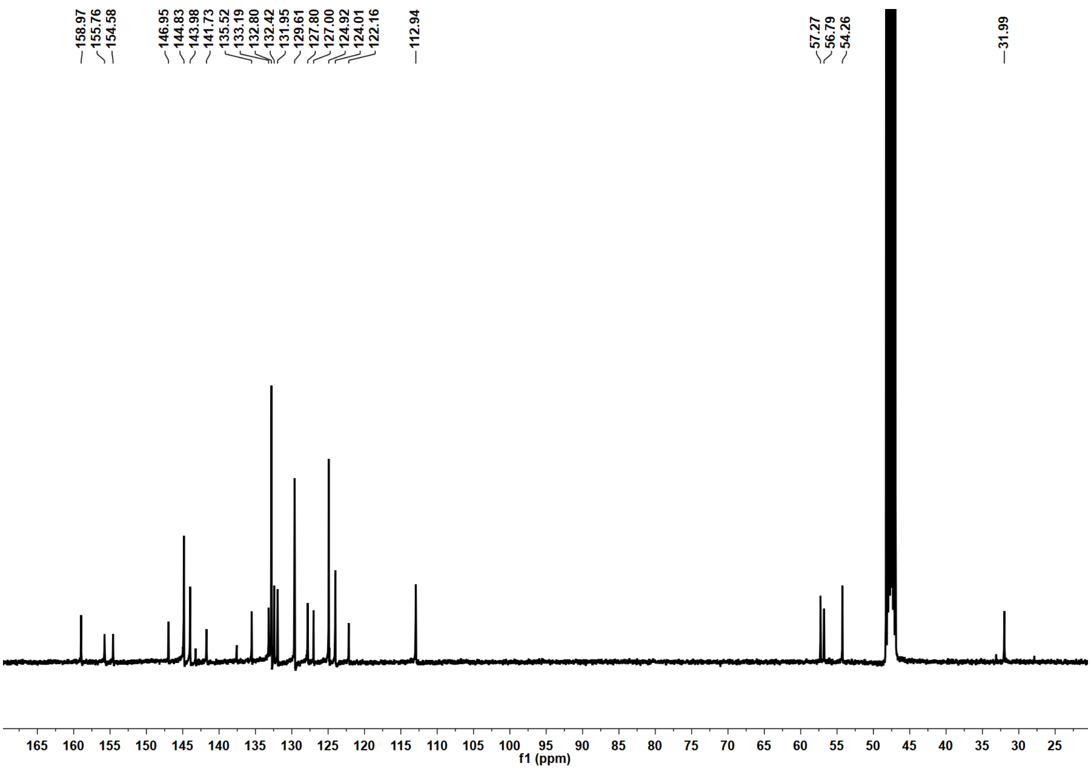

Supplementary Fig. 5 ^{13}C NMR spectrum of TPE-DPY (Methanol-*d*4, 101 MHz, 298K).

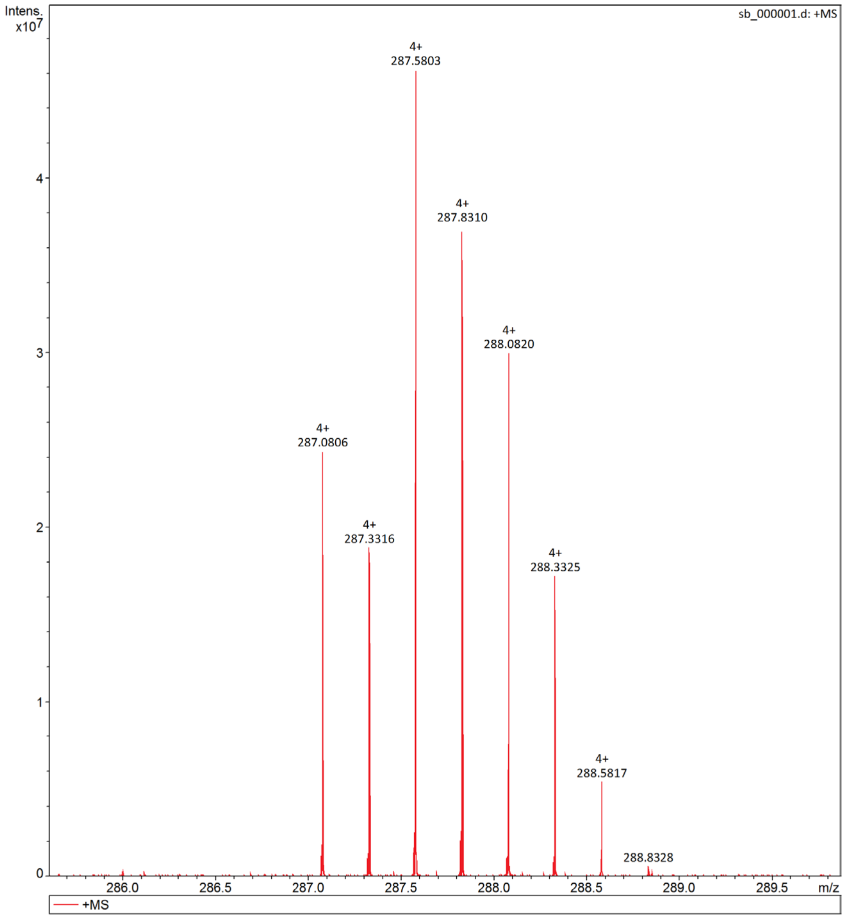

Supplementary Fig. 6 HRMS (ESI) spectrum of compound TPE-DPY.

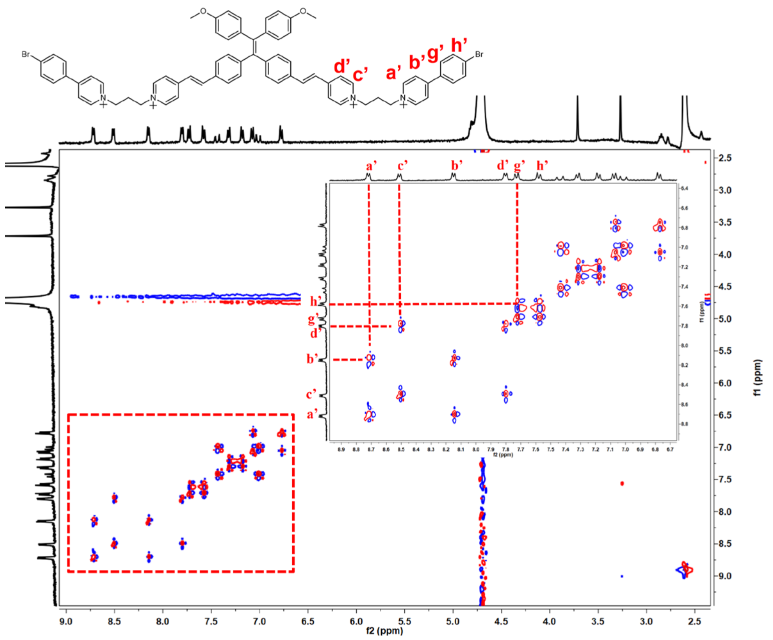

Supplementary Fig. 7 2D COSY spectrum (400 MHz, D_2O with 10% DMSO-d_6, 298 K) of TPE-DPY ([TPE-DPY] = 0.2 mM).

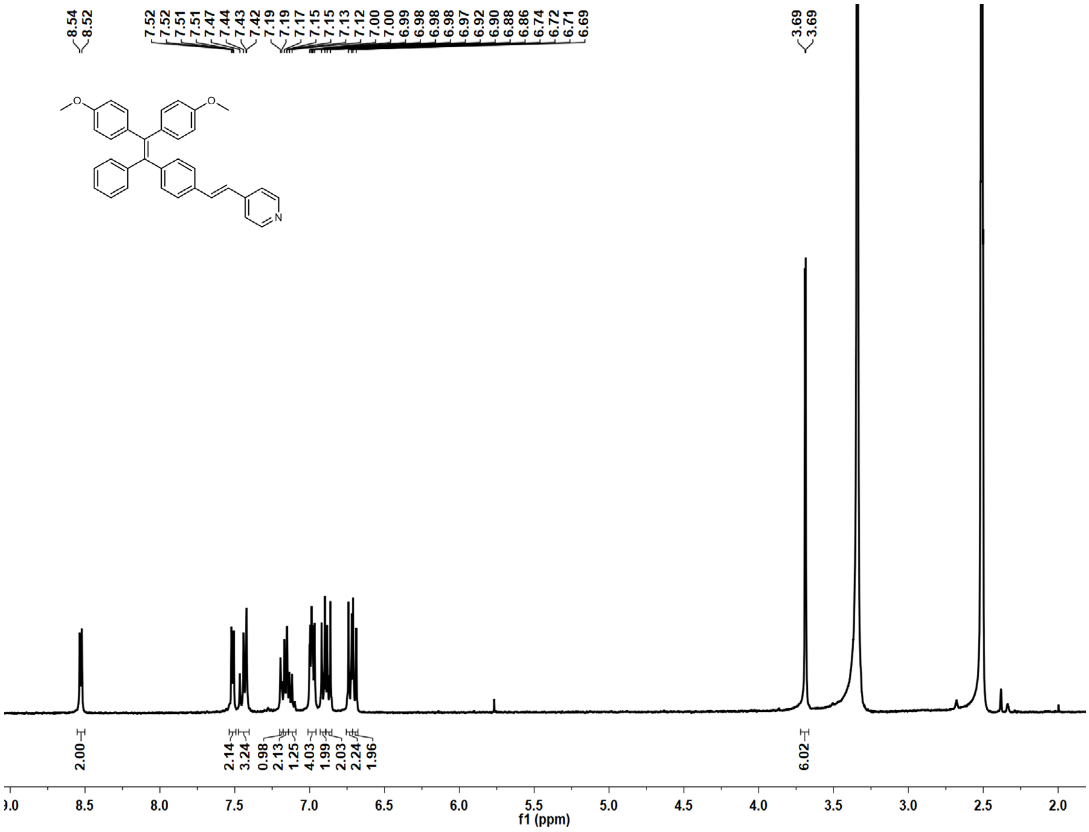

Supplementary Fig. 8 ^{1}H NMR spectrum of compound 3 (DMSO-d_6, 400 MHz, 298K).

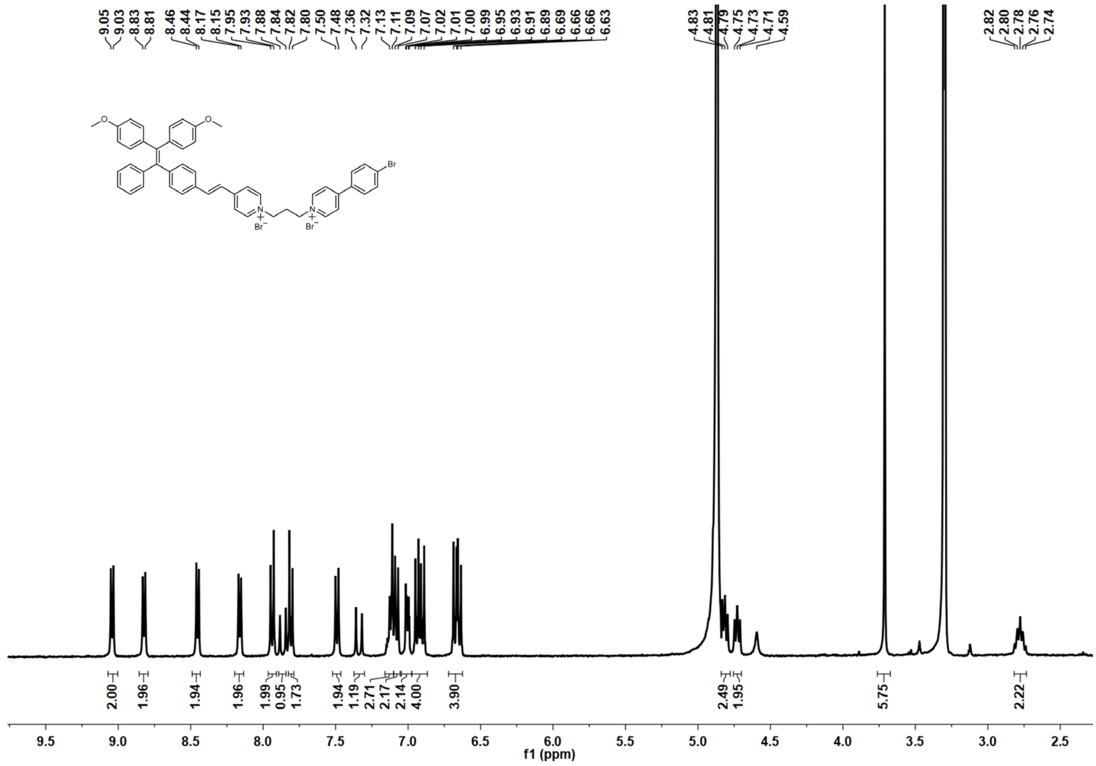

Supplementary Fig. 9 ^{1}H NMR spectrum of TPE-PY (Methanol-d_4, 400 MHz, 298K).

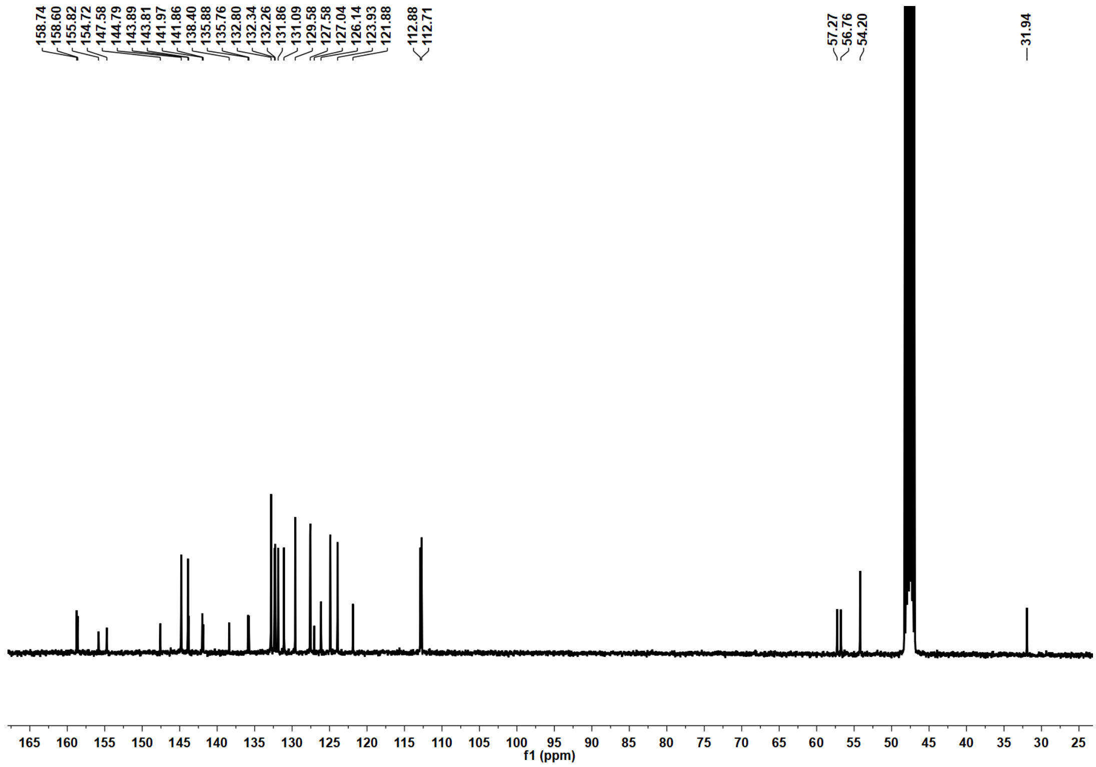

Supplementary Fig. 10 ^{13}C NMR spectrum of TPE-PY (Methanol-d_4, 101 MHz, 298K).

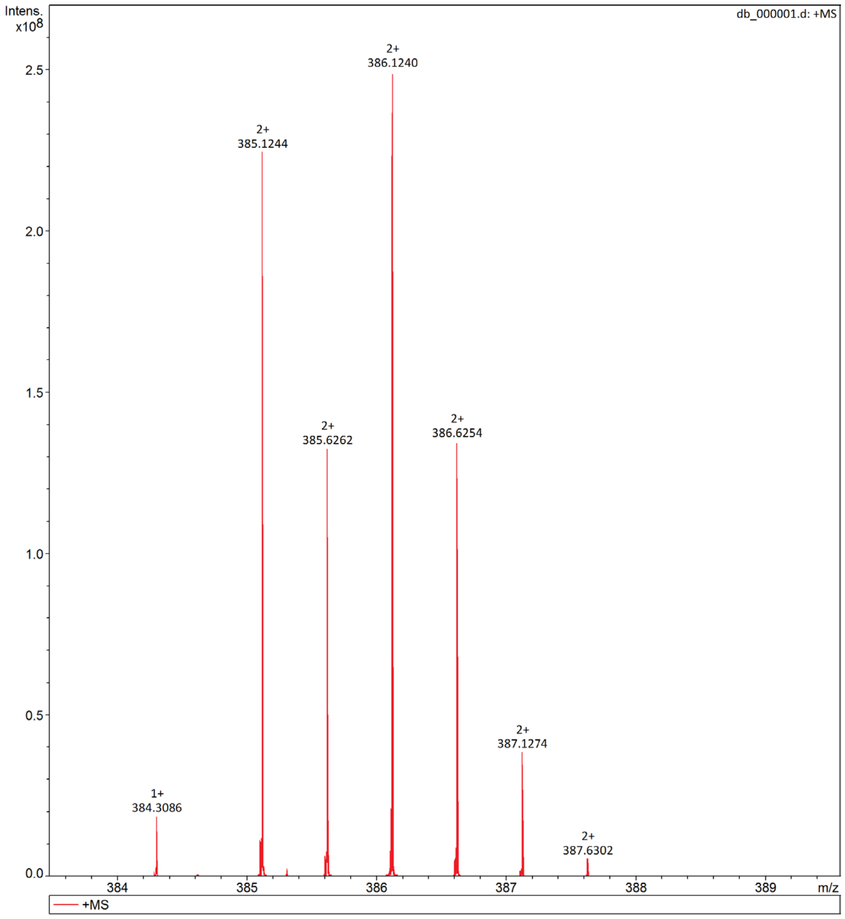

Supplementary Fig. 11 HRMS (ESI) spectrum of TPE-PY.

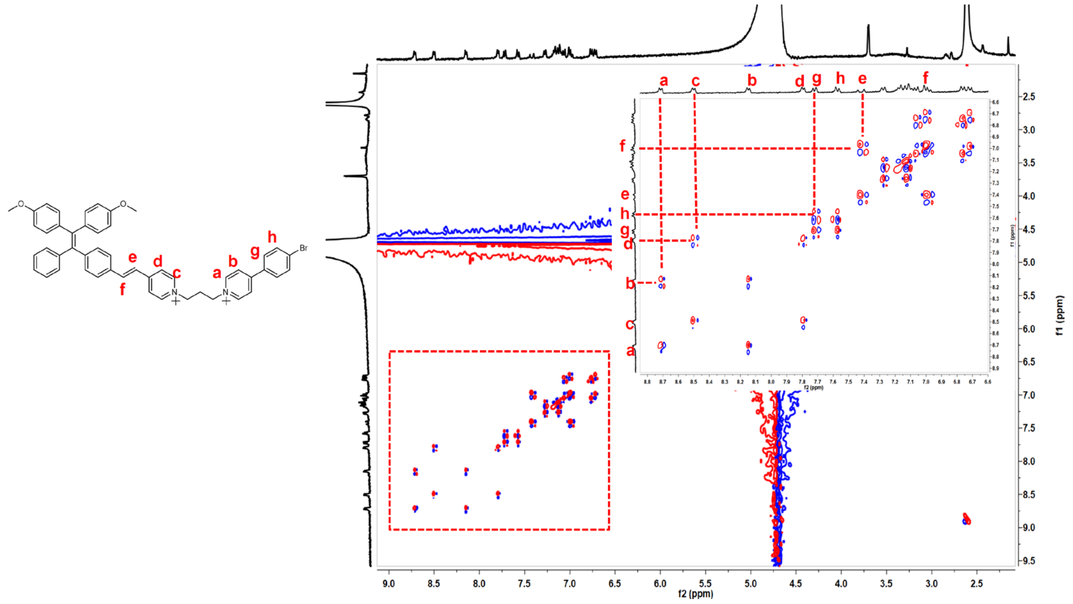

Supplementary Fig. 12 2D COSY spectrum (400 MHz, D_2O with 10% DMSO-d_6, 298 K) of TPE-PY ([TPE-PY] = 0.4 mM).

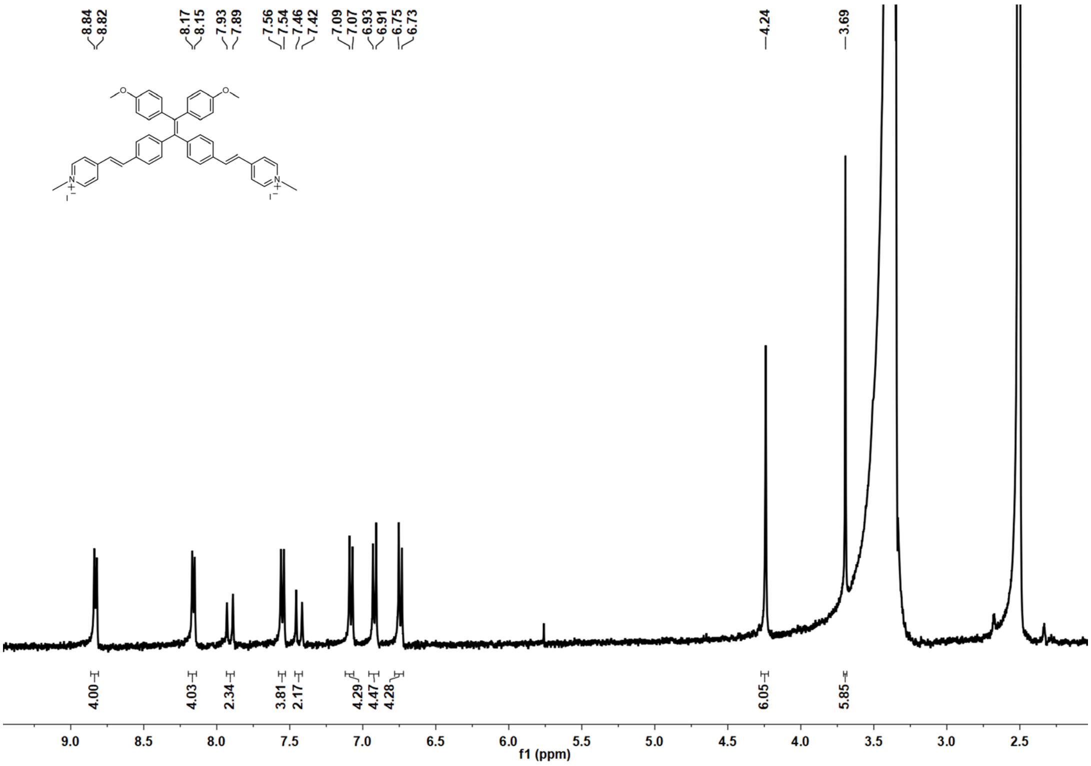

Supplementary Fig. 13 ^{1}H NMR spectrum of TPE-1 (DMSO-d_6, 400 MHz, 298K).

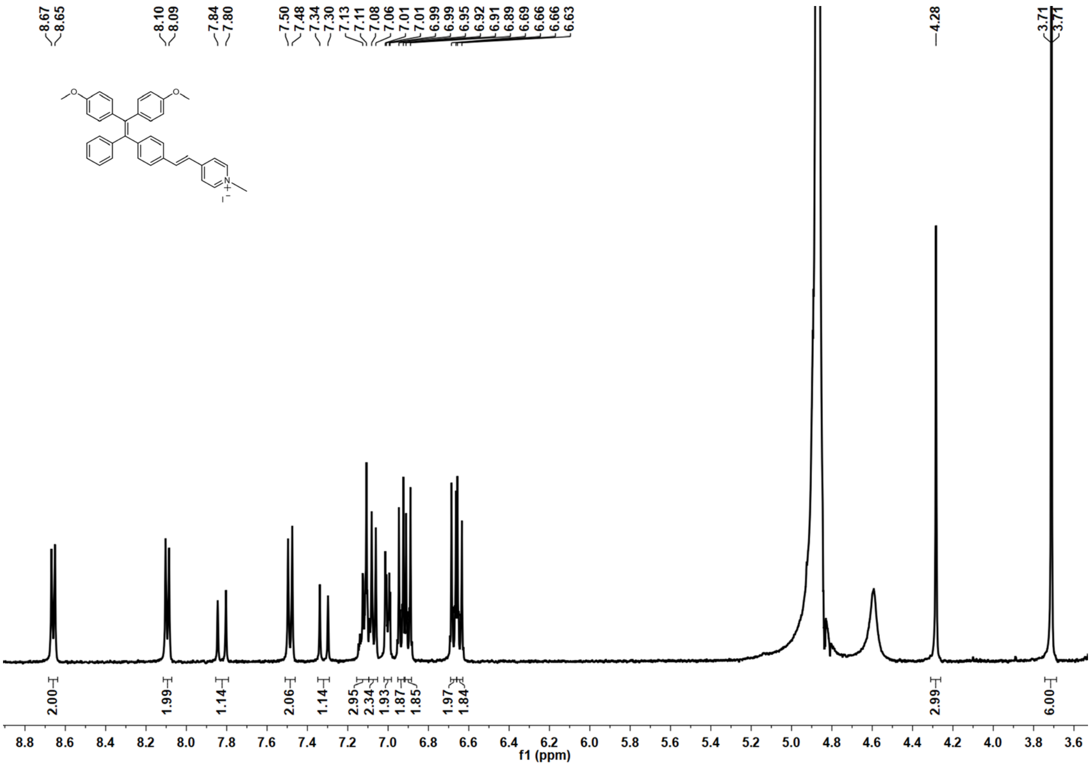

Supplementary Fig. 14 ^{1}H NMR spectrum of TPE-2 (Methanol-d_4, 400 MHz, 298K).

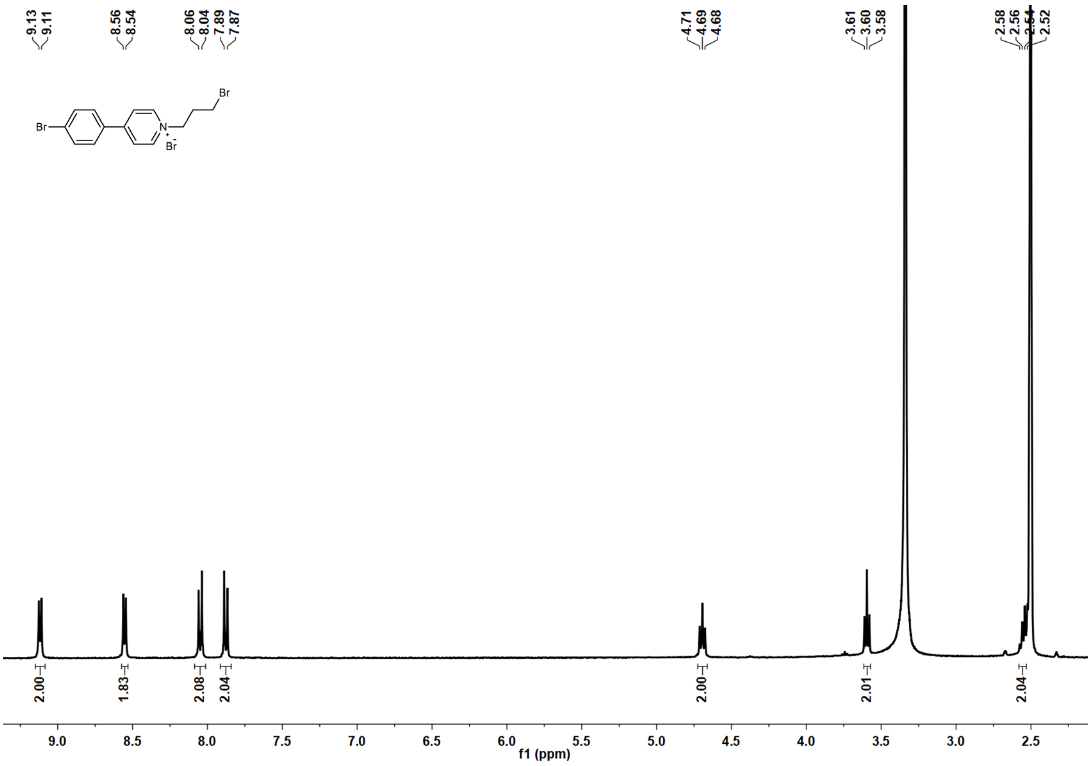

Supplementary Fig. 15 ^{1}H NMR spectrum of PY-1 (DMSO-d_6, 400 MHz, 298K).

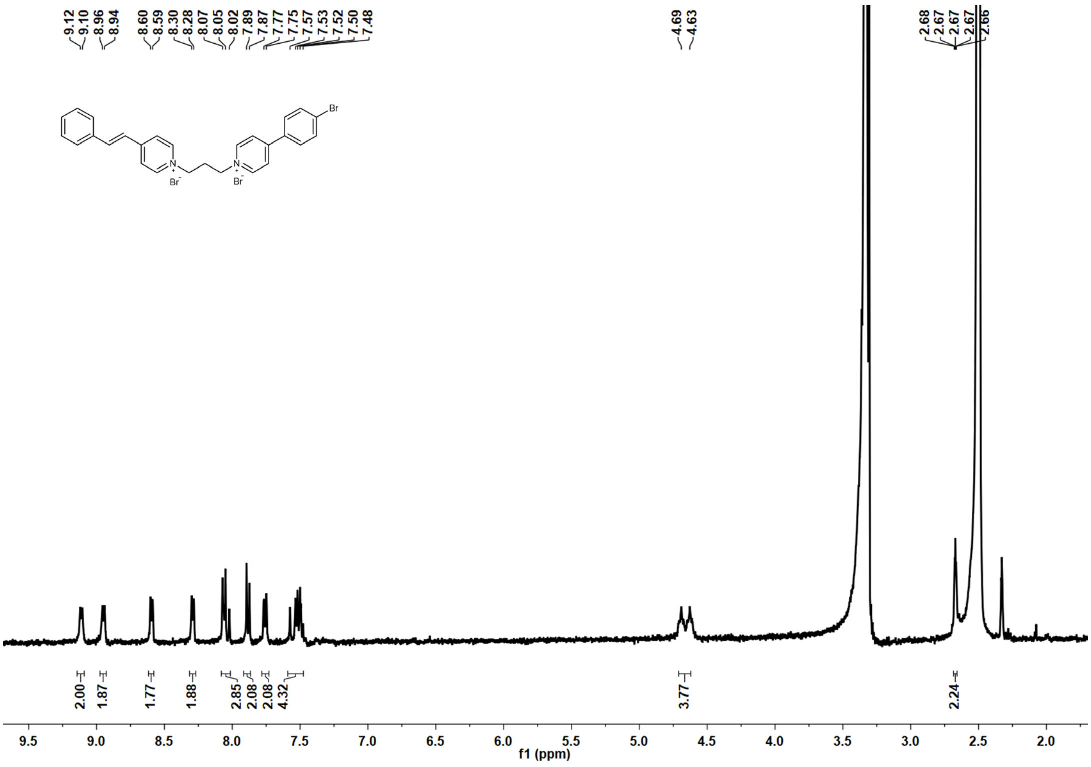

Supplementary Fig. 16 ^{1}H NMR spectrum of SP-PY (DMSO-d_6, 400 MHz, 298K).

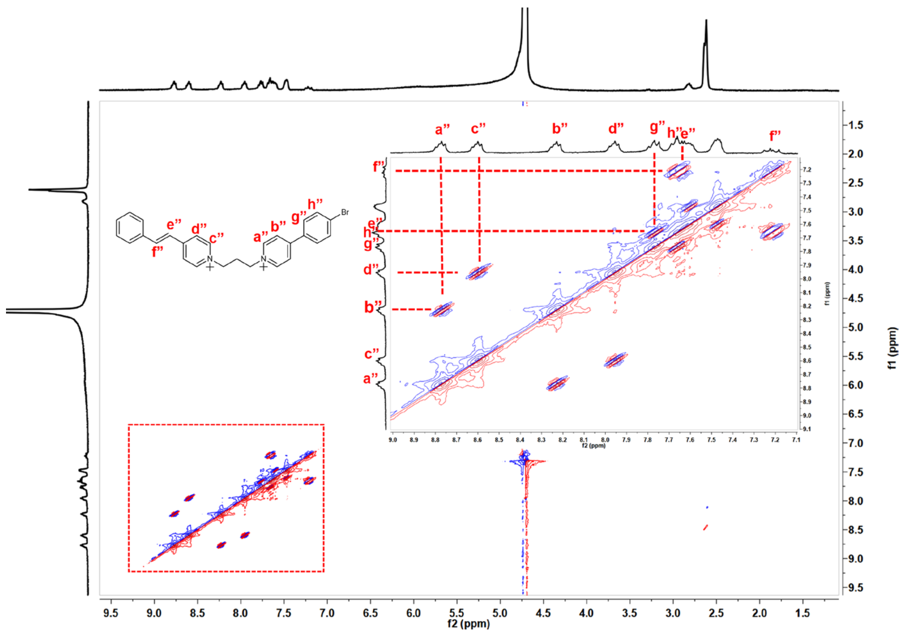

Supplementary Fig. 17 2D COSY spectrum (400 MHz, D_2O with 10% DMSO-d_6, 298 K) of SP-PY ([SP-PY] = 0.5 mM).

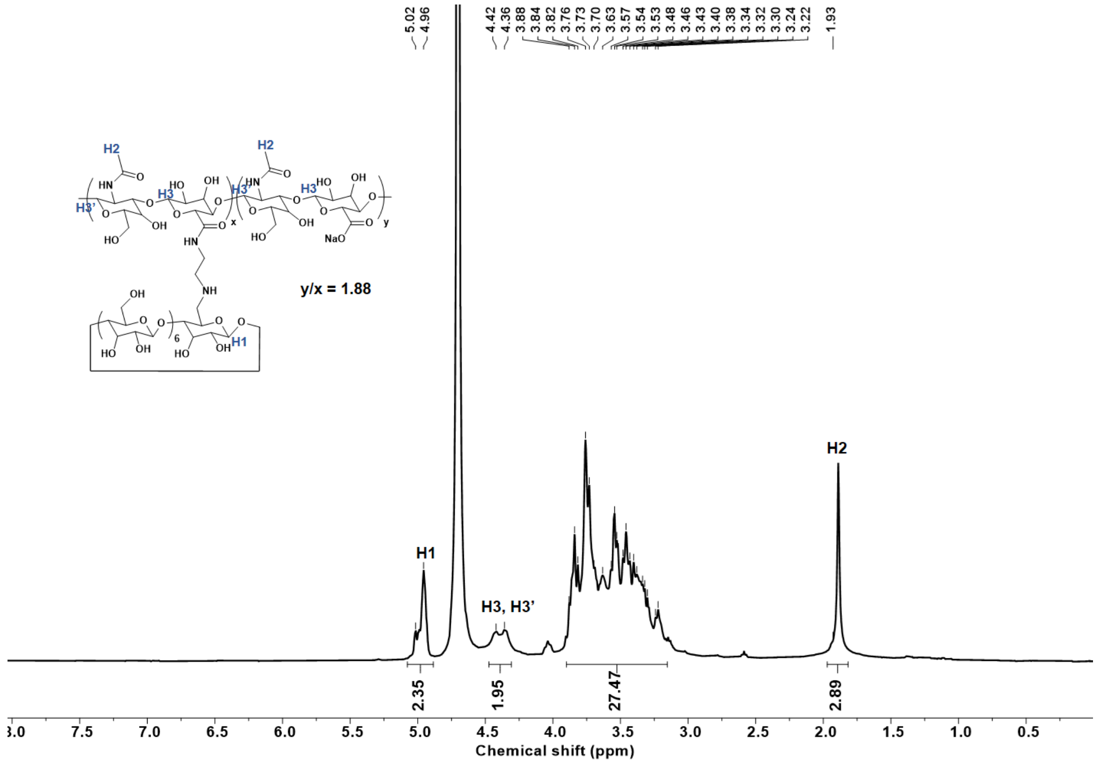

Supplementary Fig. 18 ^1H NMR spectrum of HACD (400 MHz, D_2O, 298 K).

2. Supplementary Discussion

2.1 Characterization and Photophysical Properties of Assemblies

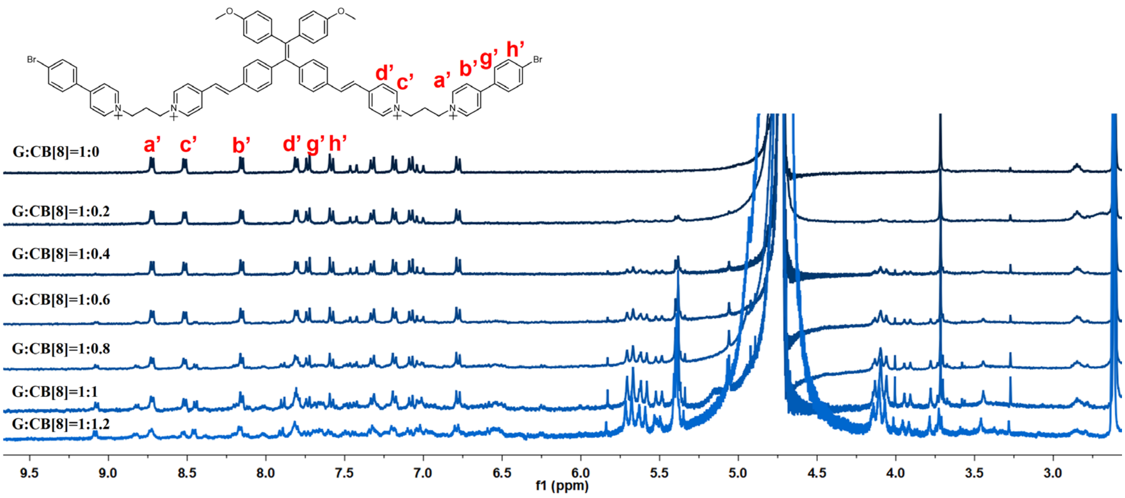

Supplementary Fig. 19 ^{1}H NMR spectral changes of TPE-DPY after adding 0, 0.2, 0.4, 0.6, 0.8, 1.0, 1.2 equivalent CB[8]. ([TPE-DPY] = 0.2 mM, 400 MHz, D_2O with 10% DMSO-d_6, 298 K).

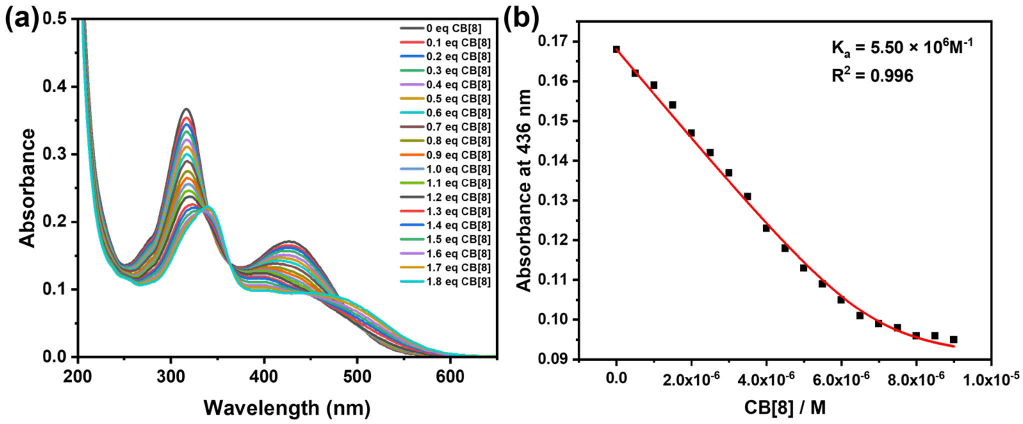

Supplementary Fig. 20 (a) The UV absorption spectra of TPE-DPY upon the addition of 0, 0.01, 0.02, …1.8 equivalent CB[8] in aqueous solution. (b) The nonlinear least-squares analyses of the UV absorbance changes with addition 0-1.8 equivalent CB[8] to calculate the association constant between TPE-DPY and CB[8] ([TPE-DPY]= 5 μM).

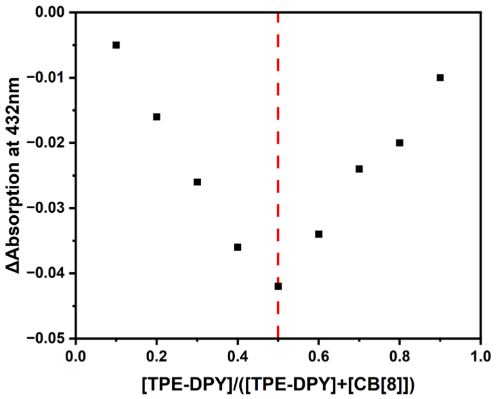

Supplementary Fig. 21 Job's plots show the 1:1 stoichiometry of the complex between TPE-DPY and CB[8] ([TPE-DPY] + [CB[8]] = 10 μM).

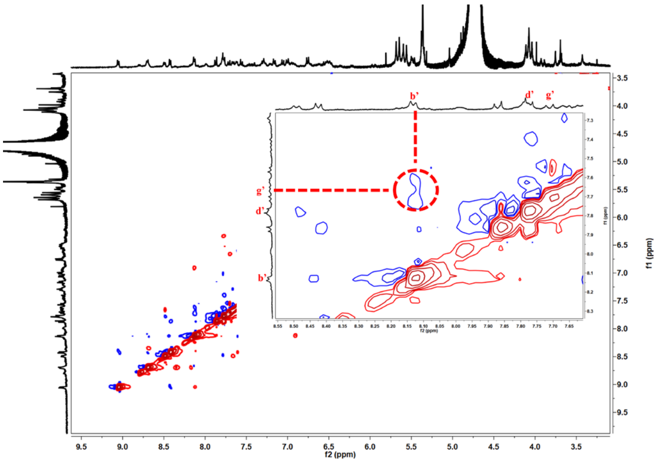

Supplementary Fig. 22 2D ROESY spectrum (400 MHz, D_2O with 10% DMSO-d_6, 298 K) of TPE-DPY in presence of 1 equivalent CB[8] ([TPE-DPY] = CB[8] = 0.2 mM).

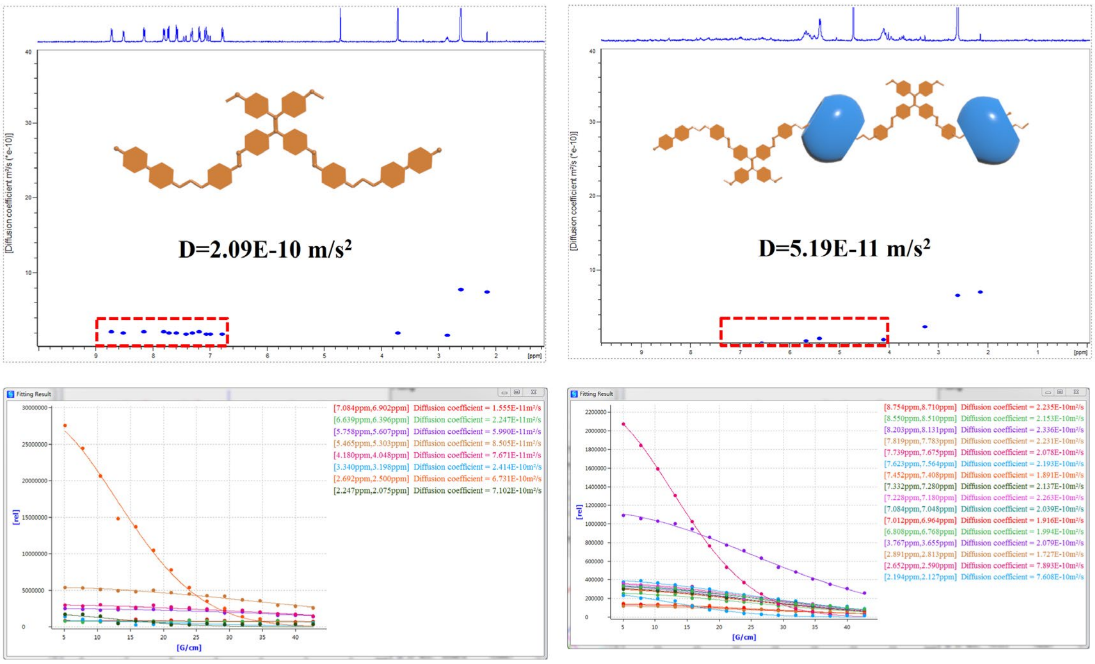

Supplementary Fig. 23 2D DOSY spectrum (400 MHz, D_2O with 10% DMSO-d_6, 298 K) of TPE-DPY, and TPE-DPY in the presence of 1 equivalent CB[8] ([TPE-DPY] = [CB[8]] = 0.2 mM).

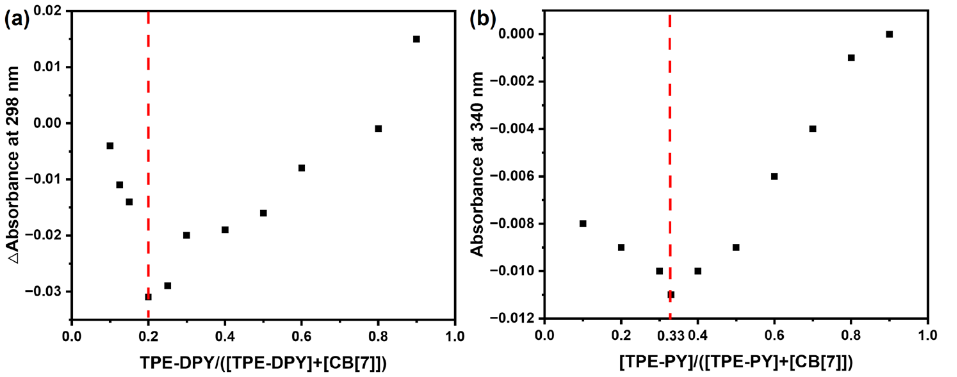

Supplementary Fig. 24 Job's experiment for TPE-DPY (a) and TPE-PY (b) upon complexation with CB[7] in aqueous solution at 298 K. Absorbance intensity changes of TPE-DPY recorded at 298 nm and TPE-PY recorded at 340 nm were used to analysis the binding ratio ([TPE-DPY] + [CB[7]] = 10 μM, [TPE-PY] + [CB[7]] = 10 μM).

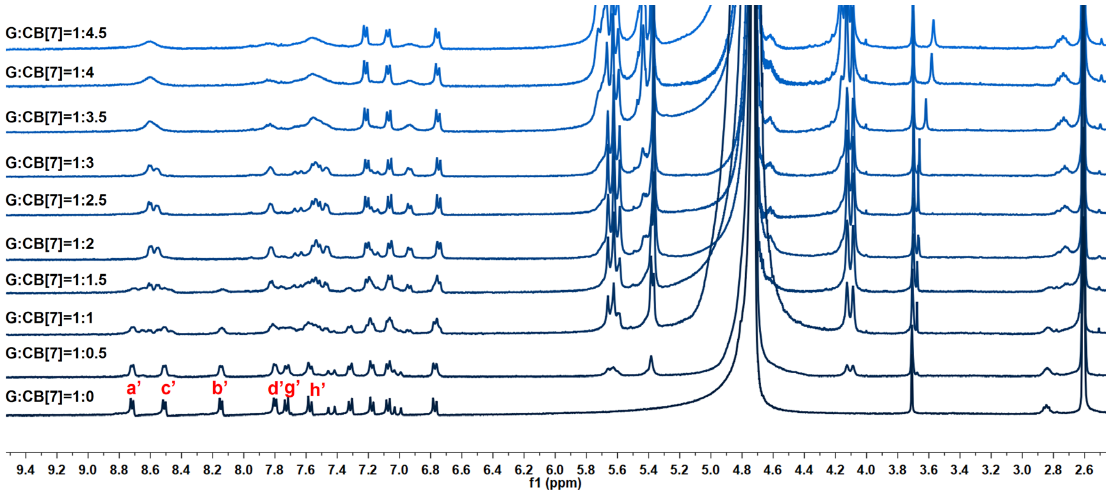

Supplementary Fig. 25 ^{1}H NMR spectra (400 MHz, D_2O with 10% DMSO-d_6, 298 K) of TPE-DPY (0.4 mM), with the gradient concentration addition of CB[7] (from 0 to 4.5 equivalent).

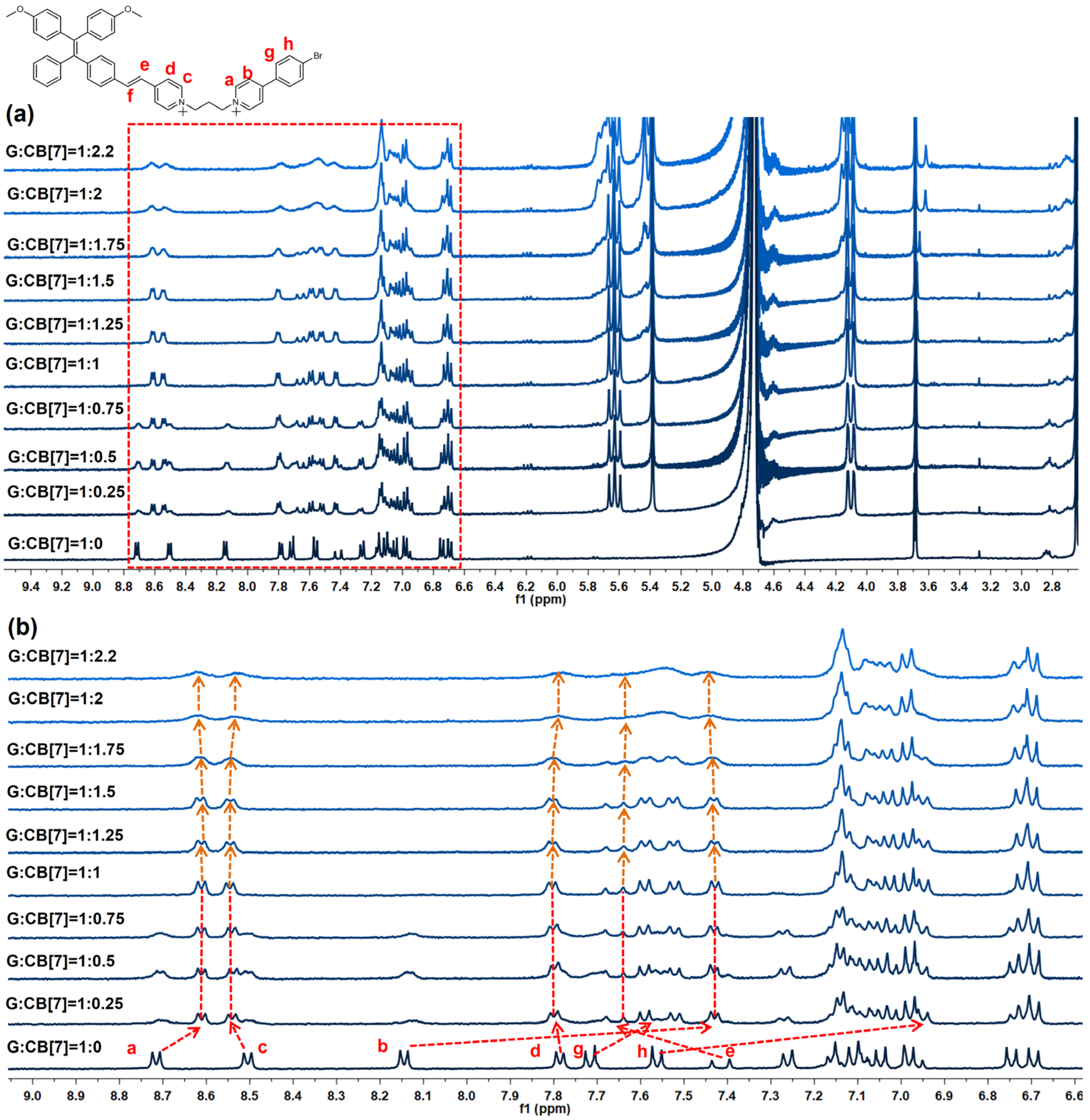

Supplementary Fig. 26 (a) ^{1}H NMR spectra (400 MHz, D_2O with 10% DMSO-d_6, 298 K) of TPE-

PY (0.4 mM), with the gradient concentration addition of CB[7] (from 0 to 2.2 equivalent), and (b) its partial enlargement of hydrogen peaks in aromatic region.

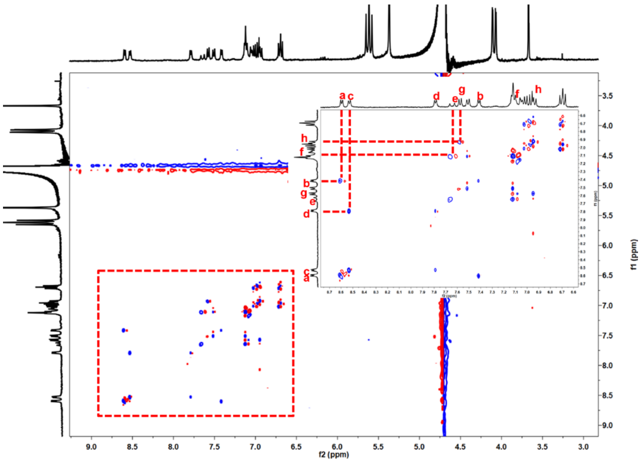

Supplementary Fig. 27 2D COSY spectrum (400 MHz, D_2O with 10% DMSO-d_6, 298 K) of TPE-PY/1CB[7] ([TPE-PY] = [CB[7]] = 0.4 mM).

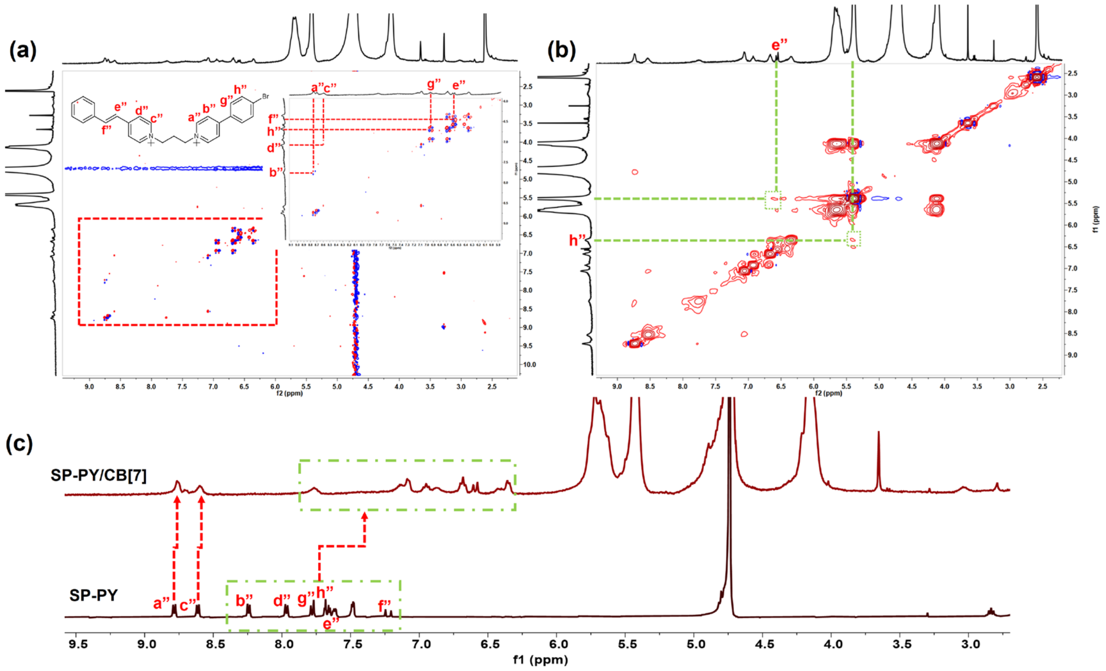

Supplementary Fig. 28 Characterization of binding behavior between SP-PY and CB[7]. (a) 2D COSY spectrum (400 MHz, D_2O with 10% DMSO-d_6, 298 K) of SP-PY/CB[7] ([CB[7]] = 2[SP-PY] = 1.0 mM). (b) 2D NOESY (400 MHz, D_2O with 10% DMSO-d_6, 298 K) of SP-PY/CB[7] ([CB[7]] = 2[SP-PY] = 1.0 mM). (c) ^1H NMR spectra (400 MHz, D_2O with 10% DMSO-d_6, 298 K) of SP-PY (0.5 mM) and SP-PY/CB[7] ([CB[7]] = 2[SP-PY] = 1.0 mM).

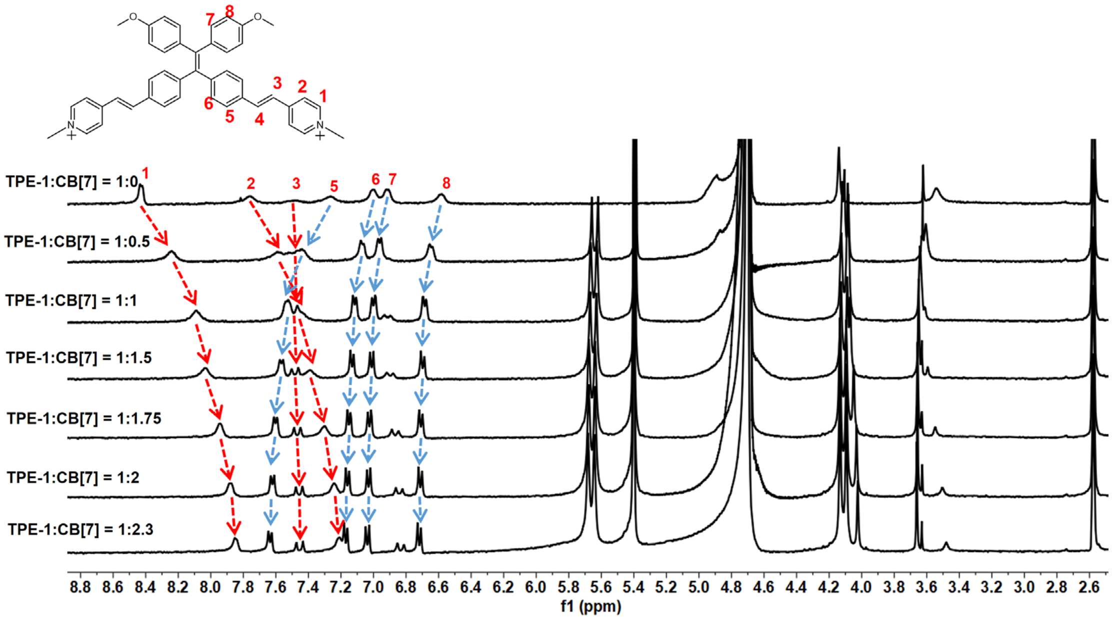

Supplementary Fig. 29 ^{1}H NMR spectra (400 MHz, D_2O with 10% DMSO-d_6, 298 K) of TPE-1 (0.4 mM), with the gradient concentration addition of CB[7] (from 0 to 2.3 equivalent).

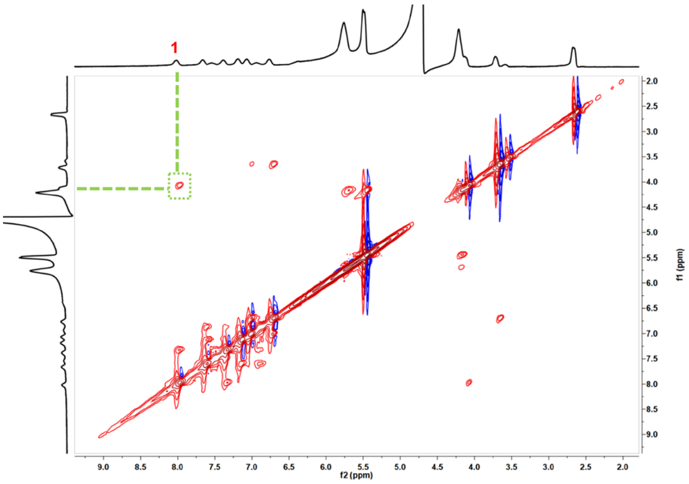

Supplementary Fig. 30 2D NOESY (400 MHz, D_2O with 10% DMSO-d_6, 298 K) of TPE-1/CB[7] ([CB[7]] = 2[TPE-1] = 0.8 mM).

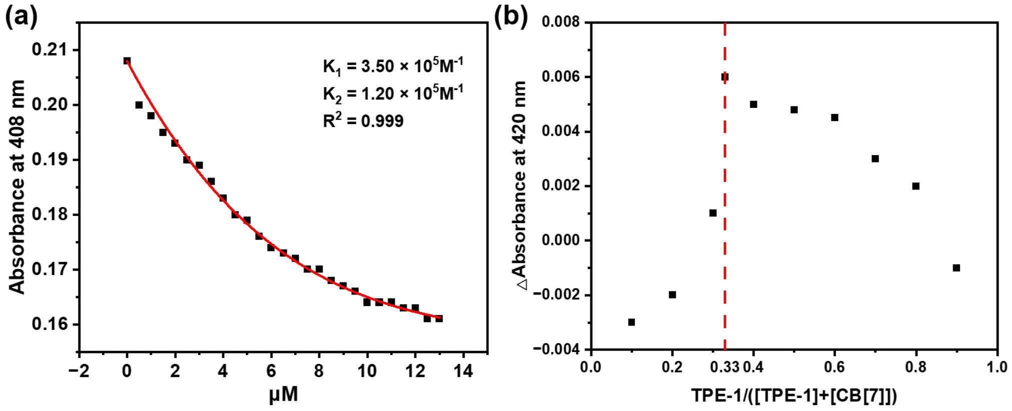

Supplementary Fig. 31 (a) The nonlinear least-squares analyses of the UV absorbance changes with addition 0-2.6 equivalent CB[7] to calculate the association constant between TPE-1 and CB[7] ([TPE-1]= 5 μM). (b) Job's experiment for TPE-1 upon complexation with CB[7] in aqueous solution at 298 K ([TPE-1] + [CB[7]] = 10 μM).

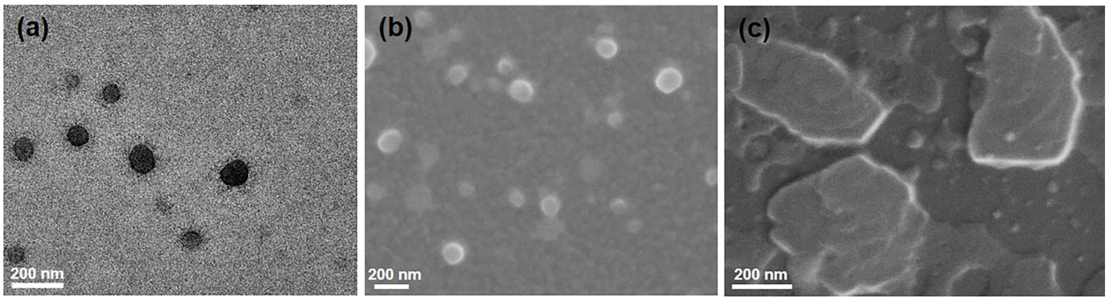

Supplementary Fig. 32 TEM (a) and SEM images (b) of TPE-DPY/2CB[7] ([TPE-DPY] = 10 μM, [CB[7]] = 20 μM). (c) Partly enlarged SEM image of TPE-DPY/CB[7]/CB[8] ([TPE-DPY] = [CB[8]] = 10 μM, [CB[7]] = 20 μM). Each experiment was performed with three replicates with similar results.

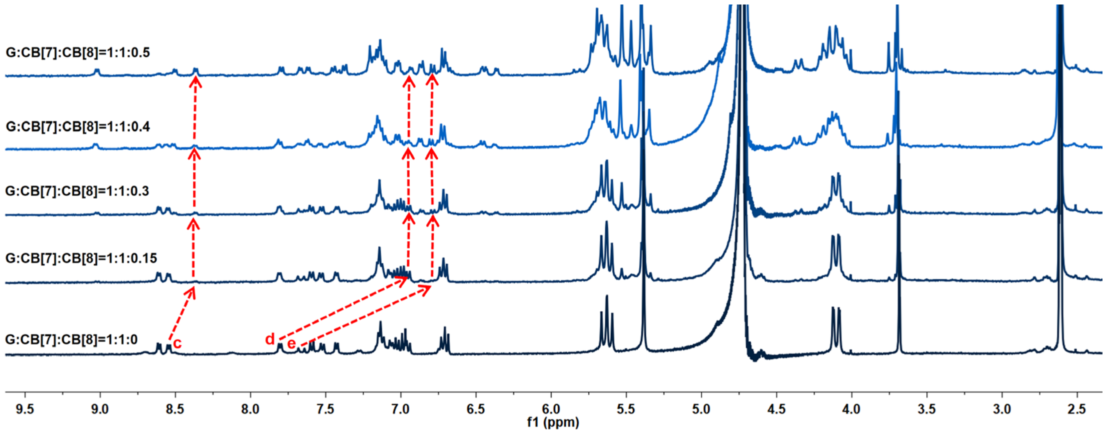

Supplementary Fig. 33 ^{1}H NMR spectra (400 MHz, D_2O with 10% DMSO-d_6, 298 K) of TPE-PY:CB[7] = 1:1 (TPE-PY] = [CB[7]] = 0.4 mM) with the gradient concentration addition of CB[8] (from 0 to 0.5 equivalent).

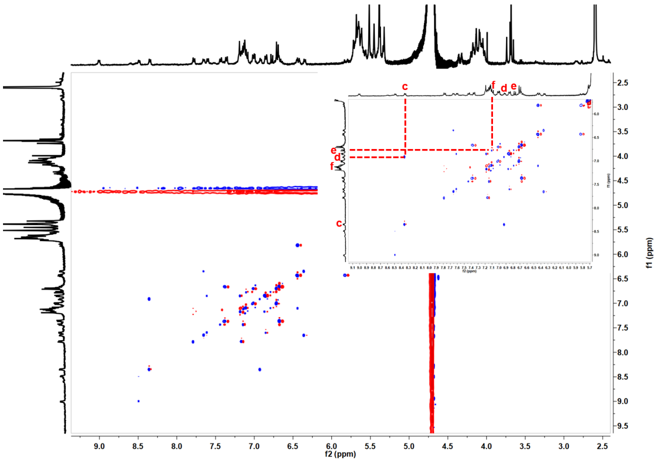

Supplementary Fig. 34 2D COSY spectrum (400 MHz, D_2O with 10% DMSO-d_6, 298 K) of TPE-PY/CB[7]/CB[8]. ([TPE-PY] = [CB[7]] = 0.4 mM; CB[8] = 0.2 mM).

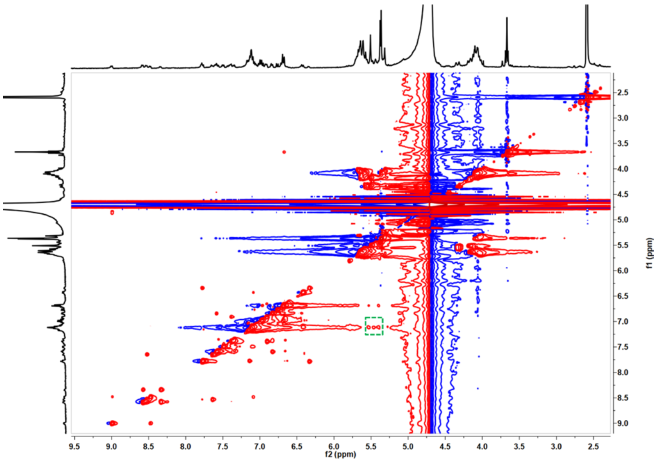

Supplementary Fig. 35 2D NOESY (400 MHz, D2O with 10% DMSO-d_6, 298 K) of TPE-PY/CB[7]/CB[8] ([TPE-PY] = [CB[7]] =0.4 mM, [CB[8]] = 0.016 mM).

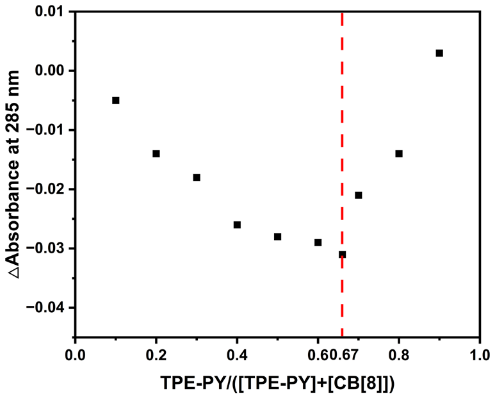

Supplementary Fig. 36 Job's plots of TPE-PY and CB[8] ([TPE-PY] + [CB[8]] = 10 μM).

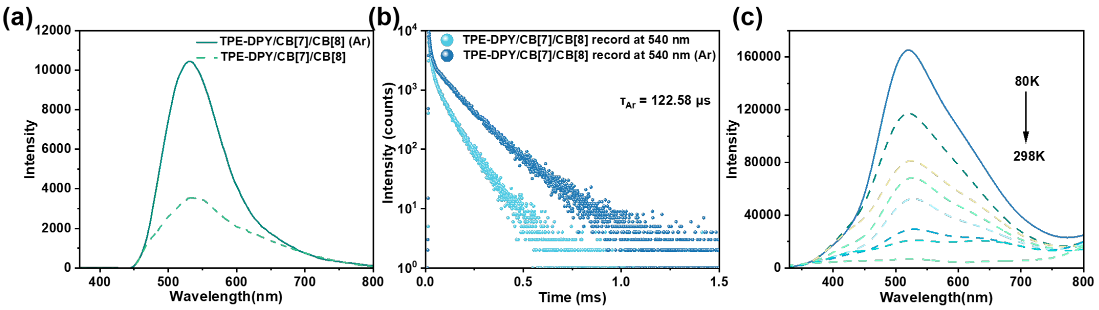

Supplementary Fig. 37 Photophysical properties of TPE-DPY/CB[7]/CB[8]. The phosphorescence spectra (a) and time-correlated decay curves (b) of TPE-DPY/CB[7]/CB[8] under air and argon atmosphere (lifetime recorded at 540 nm); (c) The temperature-dependent phosphorescence spectrum of TPE-DPY/CB[7]/CB[8] decreased from 298 K to 80 K. ([TPE-DPY] = [CB[8]] = 25 μM, [CB[7]] = 50 μM).

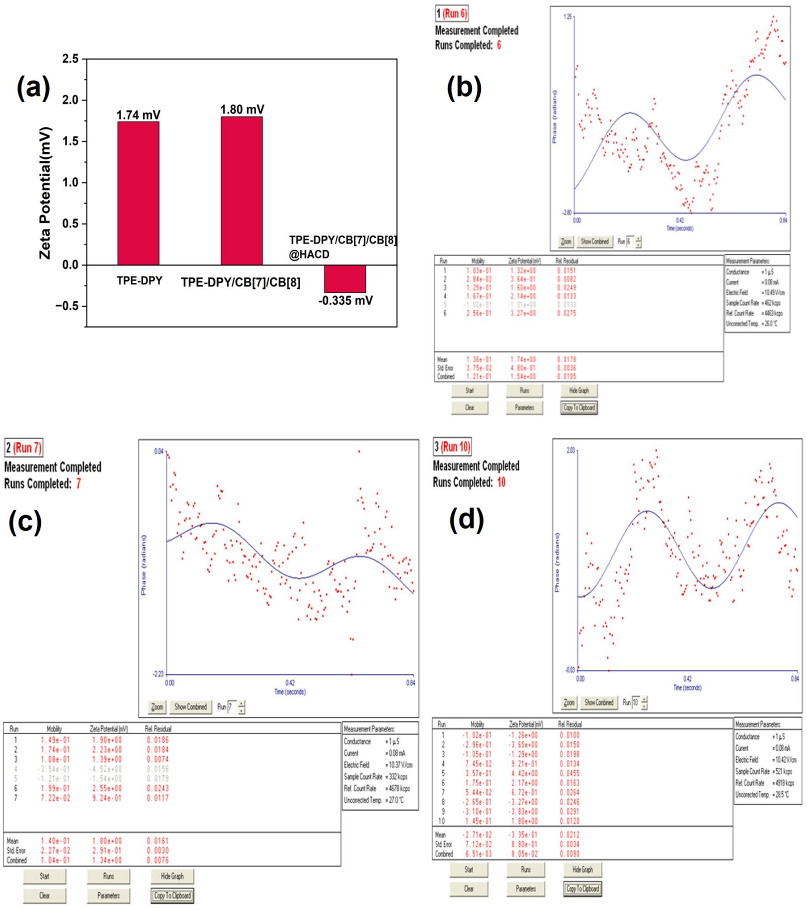

Supplementary Fig. 38 (a) Zeta potential of TPE-DPY, TPE-DPY/CB[7]/CB[8] and TPE-DPY/CB[7]/CB[8]@HACD. The original data of Zeta potential of (b) TPE-DPY, (c) TPE-DPY/CB[7]/CB[8] and (d) TPE-DPY/CB[7]/CB[8]@HACD. ([TPE-DPY] = [CB[8]] =10 μM, [CB[7]] = 20 μM, [HACD] = 0.018 mg/ml)

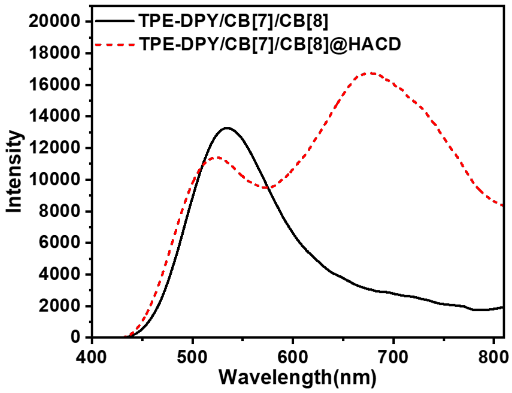

Supplementary Fig. 39 The phosphorescence spectra of TPE-DPY/CB[7]/CB[8] and TPE-DPY/CB[7]/CB[8]@HACD ([TPE-DPY] = [CB[8]] = 25 μM, [CB[7]] = 50 μM, [HACD] = 0.045 mg/ml, λ_{ex} = 333 nm).

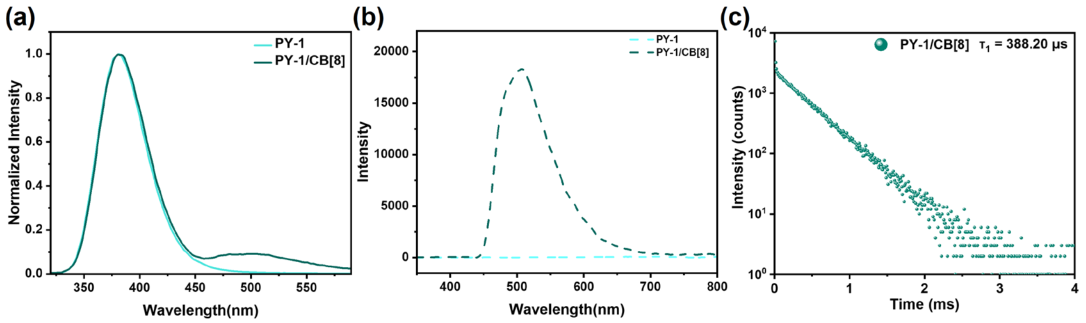

Supplementary Fig. 40 Photophysical properties of PY-1 and PY-1/CB[8]. The steady-state PL (a) and delayed spectra (b) of PY-1 and PY-1/CB[8]. (c) Time-correlated decay curve of PY-1/CB[8] at 510 nm ([PY-1] = 25 μM, [CB[8]] = 12.5 μM).

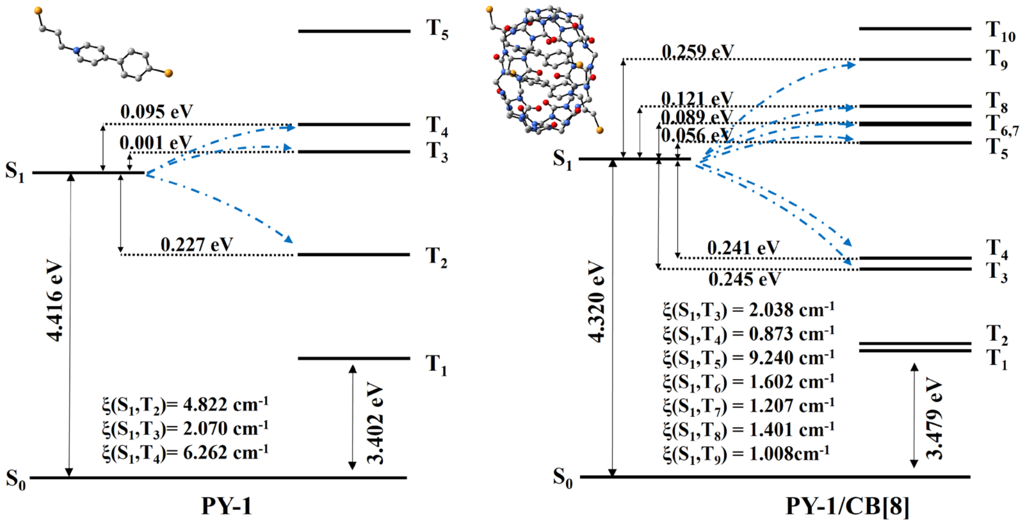

Supplementary Fig. 41 Theoretical energy level diagrams and crucial SOC coefficients in PY-1

and PY-1/CB[8].

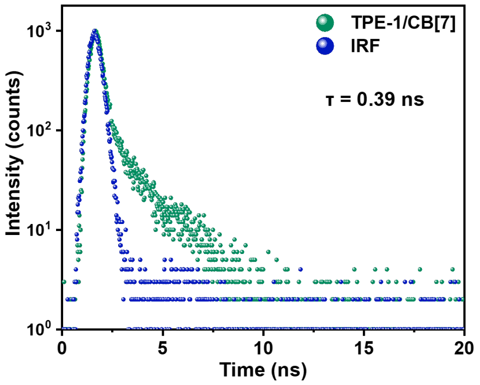

Supplementary Fig. 42 Time-correlated decay curve of TPE-1/CB[7] at 720 nm ([TPE-1] = 25 μM, [CB[7]] = 50 μM).

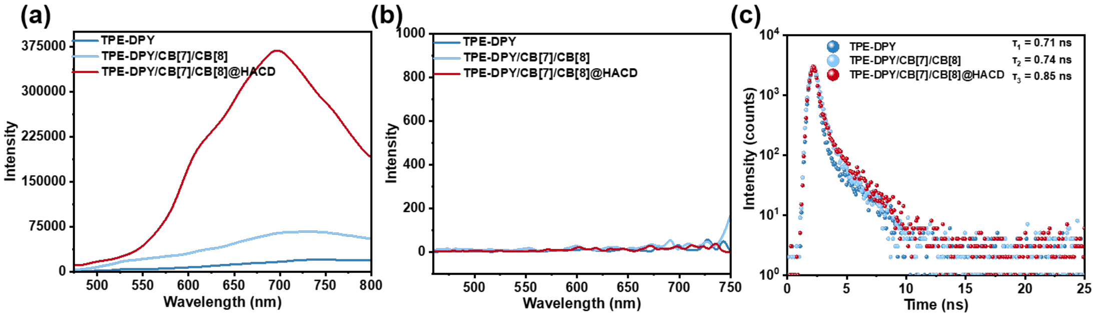

Supplementary Fig. 43 NIR fluorescence properties of TPE-DPY and the assemblies. The PL spectrum (a), delayed spectrum (b) and time-correlated decay curves (c) of TPE-DPY, TPE-DPY/CB[7]/CB[8], and TPE-DPY/CB[7]/CB[8]@HACD (λ_{ex} = 450 nm, the lifetime recorded at 720 nm, [TPE-DPY] = 25 μM, [CB[7]] = 50 μM, [CB[8]] = 25 μM, [HACD] = 0.045 mg/ml).

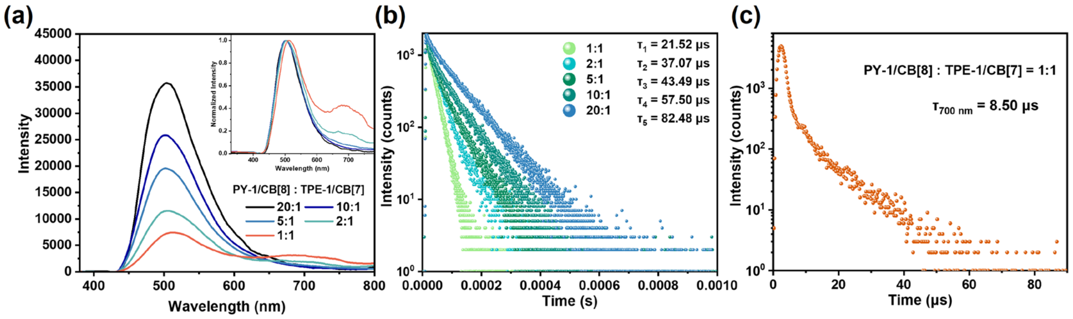

Supplementary Fig. 44 PRET phenomenon in the doping system with PY-1/CB[8] and TPE-1/CB[7]. (a) The delayed spectra and normalized delayed spectra of the doping system of PY-1/CB[8]-TPE-1/CB[7]@HACD ([PY-1] = 2[CB[8]] = 25 μM, [HACD] = 0.045 mg/ml) with

different molar ratios of TPE-1/CB[7] (delay time = 50 μs). (b) Time-correlated decay curves of PY-1/CB[8]-TPE-1/CB[7]@HACD in different doping ratios (λ_{ex} = 330 nm, the lifetime recorded at 520 nm). (c) Time-correlated decay curves of PY-1/CB[8]-TPE-1/CB[7]@HACD ([PY-1] = 2[CB[8]] = 25 μM, [TPE-1] = 0.5[CB[7]] = 25 μM, [HACD] = 0.045 mg/ml) (λ_{ex} = 330 nm, the lifetime recorded at 700 nm).

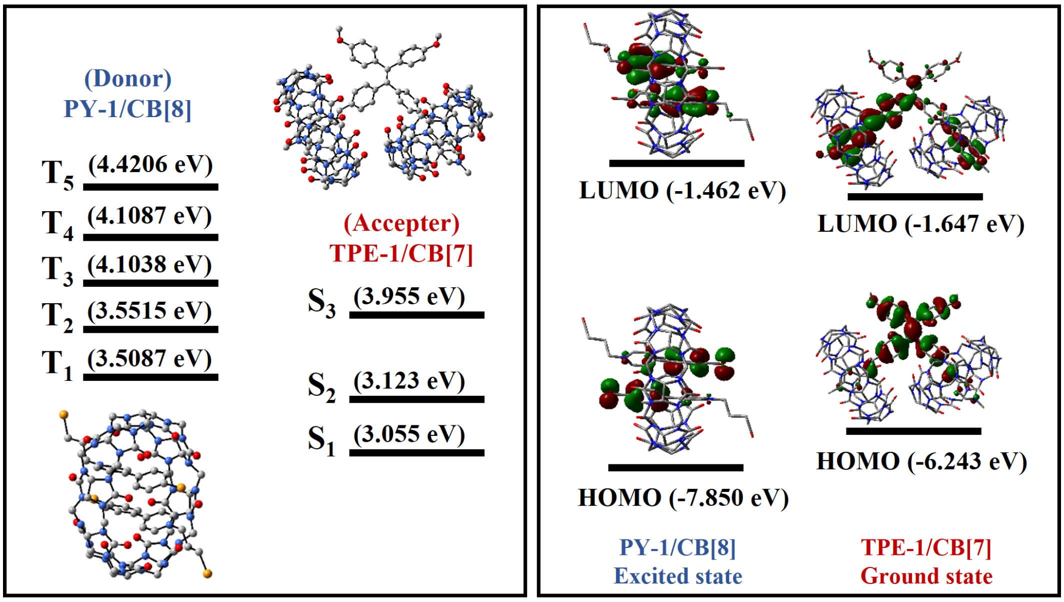

Supplementary Fig. 45 Theoretical energy level diagrams, molecular orbitals and the corresponding energies of PY-1/CB[8] and TPE-1/CB[7].

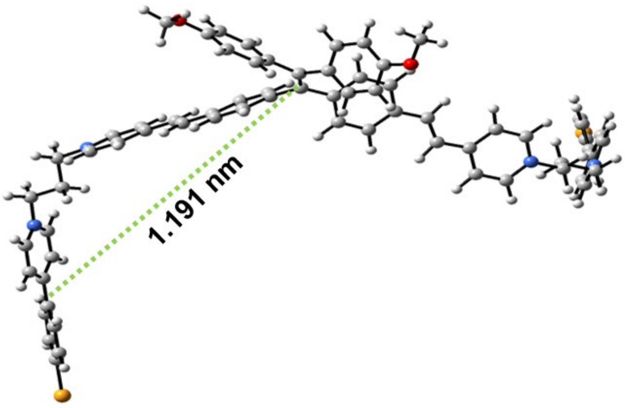

Supplementary Fig. 46 The geometrically optimized molecular structure of TPE-DPY.

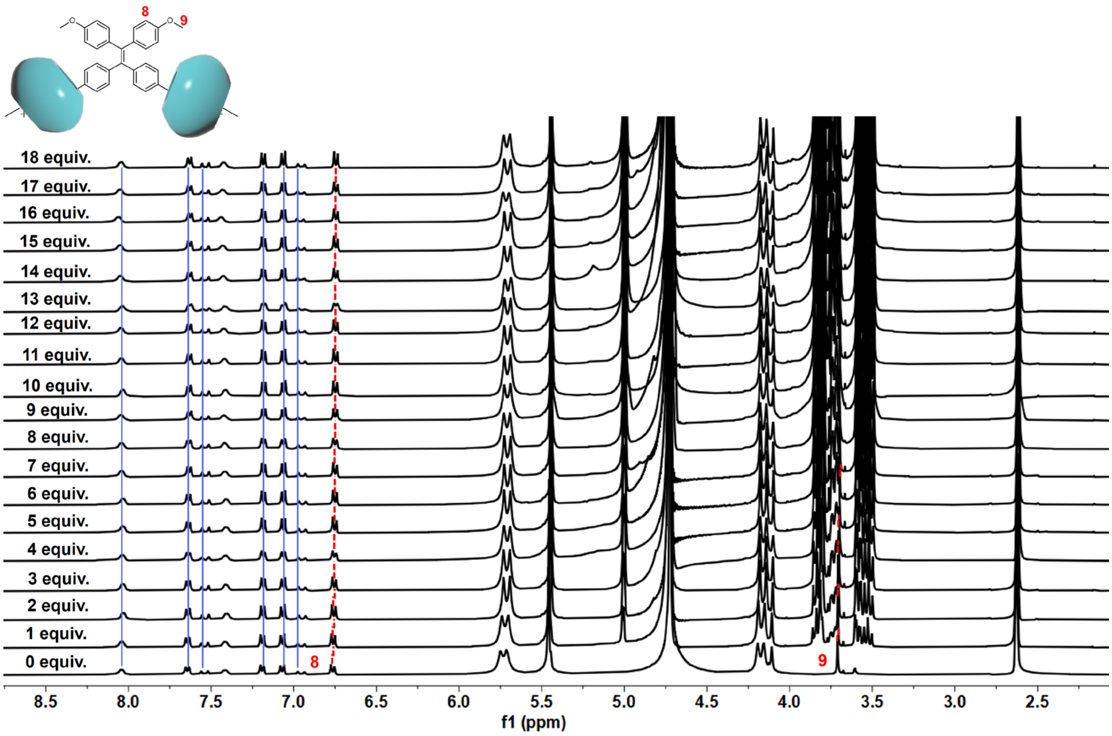

Supplementary Fig. 47 ^{1}H NMR spectral changes of TPE-1/CB[7] after adding 0, 1, 2, 3 …… 17, 18 equivalent β-CD. (CB[7] = 2[TPE-1] = 1 mM, 400 MHz, D_2O with 10% DMSO-d_6, 298 K).

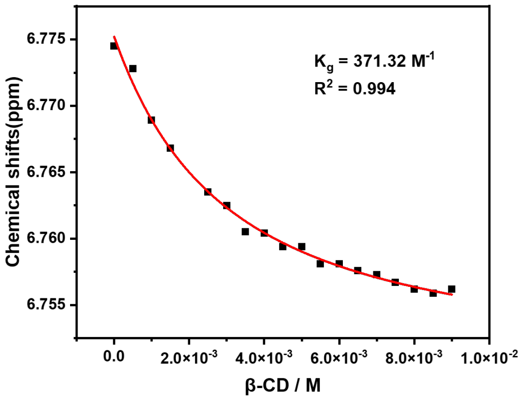

Supplementary Fig. 48 The nonlinear least-squares analyses of the chemical shift changes with addition 0, 1, 2, 3 …… 17, 18 equivalent β-CD to calculate the association constant between TPE-1/CB[7] and β-CD (CB[7] = 2[TPE-1] = 1 mM).

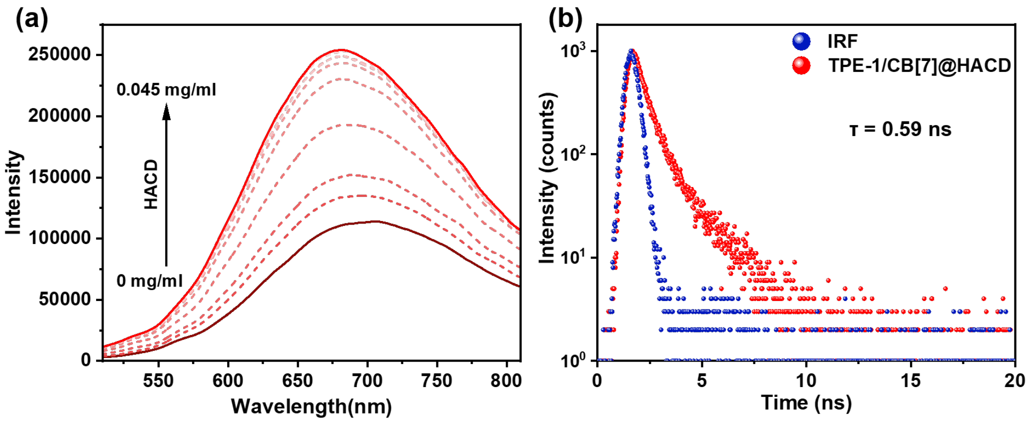

Supplementary Fig. 49 (a) PL spectrum of TPE-1/CB[7] upon adding 0-0.045 mg/ml HACD ([TPE-1] = 0.5[CB[7]] = 25 μM, λ_{ex} = 450 nm); (b) Time-correlated decay curve of TPE-1/CB[7]@HACD at 680 nm ([TPE-1] = 0.5[CB[7]] = 25 μM, [HACD] = 0.045 mg/ml).

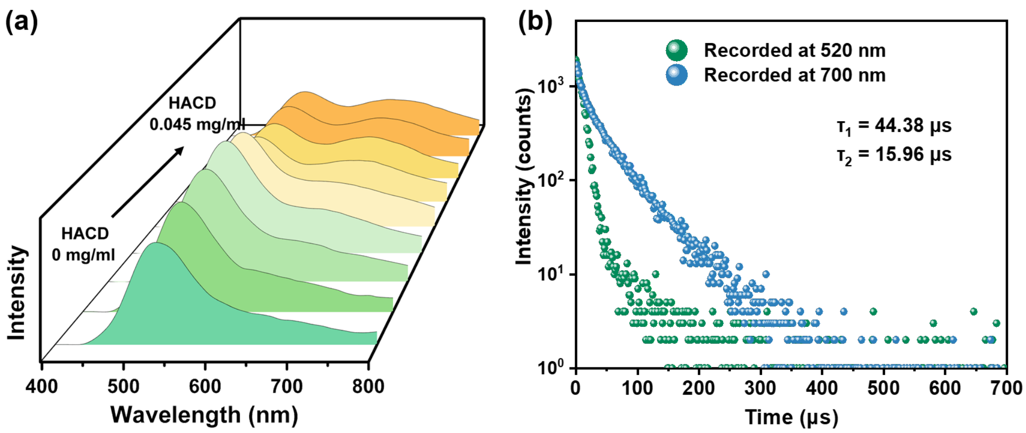

Supplementary Fig. 50 (a) Phosphorescence spectra of TPE-DPY/CB[8] upon the addition of 0-0.045 mg/ml HACD ([TPE-DPY] = [CB[8]] = 25 μM). (b) The time-resolved PL decay curves of TPE-DPY/CB[7]/CB[8]@HACD aqueous solution record at 520 nm and 700 nm.

2.2 Cells Experiments

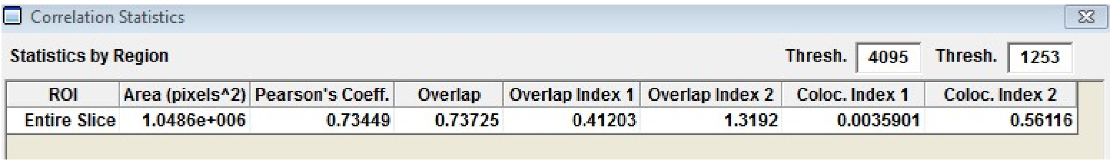

Correlation Statistics

Statistics by Region Thresh. 4095 Thresh. 1253

ROI	Area (pixels^2)	Pearson's Coeff.	Overlap	Overlap Index 1	Overlap Index 2	Coloc. Index 1	Coloc. Index 2
Entire Slice	1.0486e+006	0.73449	0.73725	0.41203	1.3192	0.0035901	0.56116

Supplementary Fig. 51 The Pearson co-efficient of merge imaging of living Hela cells in Fig. 6.

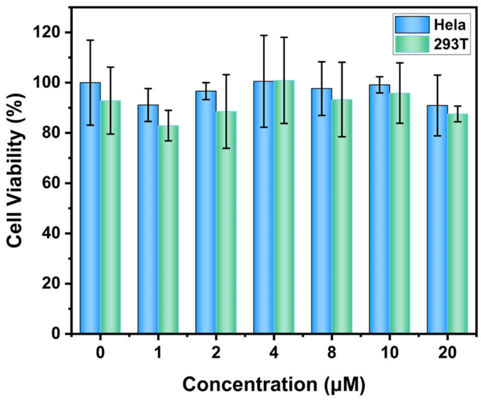

Supplementary Fig. 52 Cell viability of Hela cancer cells and 293T normal cells for TPE-DPY/CB[7]/CB[8]@HACD at different concentrations (0 μM, 1 μM, 2 μM, 4 μM, 8 μM, 10 μM, 20 μM). All bars represent mean ± SD (n = 3). ([TPE-DPY] = [CB[8]] = 20 μM, [CB[7]] = 40 μM, [HACD] = 0.036 mg/ml).

3. Supplementary References

1. Frisch, et al. Gaussian 16, Revision C.02, (Gaussian, Inc., Wallingford, CT, 2019).
2. Zhao, Y., Truhlar, D. G. The M06 Suite of Density Functionals for Main Group Thermochemistry, Thermochemical Kinetics, Noncovalent Interactions, Excited States, and Transition Elements: Two New Functionals and Systematic Testing of Four M06-Class Functionals and 12 Other Functionals. *Theor. Chem. Acc.* **120**, 215–241 (2008).
3. Grimme, S., Antony, J., Ehrlich, S., Krieg, H. A Consistent and Accurate ab Initio Parametrization of Density Functional Dispersion Correction (DFT-D) for the 94 Elements H-Pu. *J. Chem. Phys.* **132**, 154104 (2010).
4. Marenich, A. V., Cramer, C. J., Truhlar, D. G. Universal Solvation Model Based on Solute Electron Density and on a Continuum Model of the Solvent Defined by the Bulk Dielectric Constant and Atomic Surface Tensions. *J. Phys. Chem. B*. **113**, 6378–6396 (2009).
5. Weigend, F., Ahlrichs, R. Balanced Basis Sets of Split Valence, Triple Zeta Valenceand Quadruple Zeta Valence Quality for H to Rn: Design and Assessment of Accuracy. *Phys. Chem. Chem. Phys.* **7**, 3297–3305 (2005).
6. Weigend, F. Accurate Coulomb-Fitting Basis Sets for H to Rn. *Phys. Chem. Chem. Phys.* **8**, 1057–1065 (2006).
7. Neese, F., Wennmohs, F., Hansen, A., Becker, U. Efficient, Approximate and Parallel Hartree-Fock and Hybrid DFT Calculations. A 'Chain-of-Spheres' Algorithm for the Hartree-Fock Exchange. *Chem. Phys.* **356**, 98–109 (2009).
8. Neese, F. The ORCA Program System. *Wiley Interdiscip. Rev.: Comput. Mol. Sci.* **2**, 73–78 (2012).
9. Lu, T., Chen, F. Multiwfn: A Multifunctional Wavefunction Analyzer, *J. Comput. Chem.* **33**, 580-592 (2012).
10. Liu, Y. et al. Shape-Persistent π-Conjugated Macrocycles with Aggregation-Induced Emission Property: Synthesis, Mechanofluorochromism, and Mercury(II) Detection. *ACS Appl. Mater. Interfaces* **11**, 34232-34240 (2019).
11. Yang, Y., Zhang, Y.-M., Chen, Y., Chen, J.-T. & Liu, Y. Targeted Polysaccharide Nanoparticle for Adamplatin Prodrug Delivery. *J. Med. Chem*. **56**, 9725−9736 (2013).
