# Supplementary material for: Supramolecular assembly activated single-molecule phosphorescence resonance energy transfer for near-infrared targeted cell imaging
Source: Nat Commun. 2024 Jun 5;15:4787. doi: 10.1038/s41467-024-49238-5 (PMC11153566; doi:10.1038/s41467-024-49238-5)
Supplement: Supplementary file 3 — Description of Additional Supplementary Files [file 41467_2024_49238_MOESM3_ESM.pdf]

Description of Additional Supplementary Files

File name: Supplementary Data 1
Description: The atomic coordinates of the optimized computational models.
