# Supplementary material for: Supramolecular assembly activated single-molecule phosphorescence resonance energy transfer for near-infrared targeted cell imaging
Source: Nat Commun. 2024 Jun 5;15:4787. doi: 10.1038/s41467-024-49238-5 (PMC11153566; doi:10.1038/s41467-024-49238-5)
Supplement: Supplementary file 5 — Reporting Summary [file 41467_2024_49238_MOESM5_ESM.pdf]

nature portfolio

Corresponding author(s): Yu Liu

Last updated by author(s): May 16, 2024

Reporting Summary

Nature Portfolio wishes to improve the reproducibility of the work that we publish. This form provides structure for consistency and transparency in reporting. For further information on Nature Portfolio policies, see our Editorial Policies and the Editorial Policy Checklist.

Statistics

For all statistical analyses, confirm that the following items are present in the figure legend, table legend, main text, or Methods section.

n/a	Confirmed	
☐	☒	The exact sample size (*n*) for each experimental group/condition, given as a discrete number and unit of measurement
☐	☒	A statement on whether measurements were taken from distinct samples or whether the same sample was measured repeatedly
☐	☒	The statistical test(s) used AND whether they are one- or two-sided<br>*Only common tests should be described solely by name; describe more complex techniques in the Methods section.*
☐	☒	A description of all covariates tested
☒	☐	A description of any assumptions or corrections, such as tests of normality and adjustment for multiple comparisons
☐	☒	A full description of the statistical parameters including central tendency (e.g. means) or other basic estimates (e.g. regression coefficient) AND variation (e.g. standard deviation) or associated estimates of uncertainty (e.g. confidence intervals)
☐	☒	For null hypothesis testing, the test statistic (e.g. *F*, *t*, *r*) with confidence intervals, effect sizes, degrees of freedom and *P* value noted<br>*Give P values as exact values whenever suitable.*
☒	☐	For Bayesian analysis, information on the choice of priors and Markov chain Monte Carlo settings
☒	☐	For hierarchical and complex designs, identification of the appropriate level for tests and full reporting of outcomes
☒	☐	Estimates of effect sizes (e.g. Cohen's *d*, Pearson's *r*), indicating how they were calculated

Our web collection on statistics for biologists contains articles on many of the points above.

Software and code

Policy information about availability of computer code

Data collection	Bruker AV400, Bruker AVANCE III HD 400, Shimadzu UV-3600, Q-TOF LC-MS , Edinburgh Instruments F900, FEI Tecnai G2 F20 under 200 KV, SEM FEI Apreo S LoVac, Brookhaven ZETAPALS/BI-200SM, DLS BI-200SM, Olympus FV1000, Varioskan LUX, Gaussian 16 Revision C.02, DFT and TDDFT calculation (M062X/6-31G*),
Data analysis	Microsoft Excel 2021, OriginPro 2020b(Learning Edition), ChemDraw 20.1, MestReNova.lnk, GaussView 6.0, Multiwfn 3.8.

For manuscripts utilizing custom algorithms or software that are central to the research but not yet described in published literature, software must be made available to editors and reviewers. We strongly encourage code deposition in a community repository (e.g. GitHub). See the Nature Portfolio guidelines for submitting code & software for further information.

Data

Policy information about availability of data

All manuscripts must include a data availability statement. This statement should provide the following information, where applicable:

- Accession codes, unique identifiers, or web links for publicly available datasets
- A description of any restrictions on data availability
- For clinical datasets or third party data, please ensure that the statement adheres to our policy

The authors declare that the data supporting the findings of this study are available within the paper and its supplementary information files. Extra data are available from the corresponding author upon request. Source data are provided with this paper.

Research involving human participants, their data, or biological material

Policy information about studies with human participants or human data. See also policy information about sex, gender (identity/presentation), and sexual orientation and race, ethnicity and racism.

Reporting on sex and gender	There are no reports on sex and gender-based analyses in this paper.
Reporting on race, ethnicity, or other socially relevant groupings	There are no reports on race, ethnicity, or other socially relevant groupings in this paper.
Population characteristics	The study did not involve human research.
Recruitment	The study did not involve human research.
Ethics oversight	The study did not involve human studies or other animal experiments.

Note that full information on the approval of the study protocol must also be provided in the manuscript.

Field-specific reporting

Please select the one below that is the best fit for your research. If you are not sure, read the appropriate sections before making your selection.

[x] Life sciences [ ] Behavioural & social sciences [ ] Ecological, evolutionary & environmental sciences

For a reference copy of the document with all sections, see nature.com/documents/nr-reporting-summary-flat.pdf

Life sciences study design

All studies must disclose on these points even when the disclosure is negative.

Sample size	All cell culture experiments were performed repeatedly two times to allow for calculation of standard deviation standard errors of the mean and statistics for use in two-side Student's test. The Hela cells and 293T cells were seeded in confocal petri dishes at a density of 30000 cells per well in 1mL of complete culture medium, and we selected several different regions of the confocal dish for confocal imaging experiments. Each experiment was performed with two replicates. Each measurement was taken from two distinct samples which provide a rationale for sufficient sample sizes.
Data exclusions	No data were excluded from the analysis.
Replication	All experiments were performed with at least two technical replicates on more than one occasion to ensure reproducibility across experiments. All attempts to repeat the experiment were successful.
Randomization	Randomization was not relevant to our study design as we investigated single factors within each study.
Blinding	Blinding was not relevant as all processing methods were done with consistent parameters utilized across all treatment groups.

Reporting for specific materials, systems and methods

We require information from authors about some types of materials, experimental systems and methods used in many studies. Here, indicate whether each material, system or method listed is relevant to your study. If you are not sure if a list item applies to your research, read the appropriate section before selecting a response.

Materials & experimental systems

n/a	Involved in the study	
[x]	[ ]	Antibodies
[ ]	[x]	Eukaryotic cell lines
[x]	[ ]	Palaeontology and archaeology
[x]	[ ]	Animals and other organisms
[x]	[ ]	Clinical data
[x]	[ ]	Dual use research of concern
[x]	[ ]	Plants

Methods

n/a	Involved in the study	
[x]	[ ]	ChIP-seq
[x]	[ ]	Flow cytometry
[x]	[ ]	MRI-based neuroimaging

Eukaryotic cell lines

Policy information about cell lines and Sex and Gender in Research

Cell line source(s)	The Hela (ATCC, CCL-2) cell line and 293T (ATCC, CRL-3216) cell line, were all gained from the Cell Resource Center of China Academy of Medical Science in Beijing.
Authentication	No extra authentication was performed for the cell lines directly gained from the Cell Resource Center of China Academy of Medical Science.
Mycoplasma contamination	All cell lines tested negative for mycoplasma contamination.
Commonly misidentified lines (See ICLAC register)	No commonly misidentified lines were used.

Plants

Seed stocks	No seeds or plants were studied in this paper.
Novel plant genotypes	There is no plant research in this paper.
Authentication	There is no plant research in this paper.
